# Meta-analytic evidence on the efficacy of hypnosis for mental and somatic health issues: a 20-year perspective

**DOI:** 10.3389/fpsyg.2023.1330238

**Published:** 2024-01-08

**Authors:** Jenny Rosendahl, Cameron T. Alldredge, Antonia Haddenhorst

**Affiliations:** ^1^Institute of Psychosocial Medicine, Psychotherapy and Psychooncology, Jena University Hospital, Friedrich-Schiller-University, Jena, Germany; ^2^Department of Psychology and Neuroscience, Baylor University, Waco, TX, United States

**Keywords:** hypnosis, hypnotherapy, meta-analysis, randomized controlled trial, efficacy

## Abstract

**Introduction:**

Documented use and investigation of hypnosis spans centuries and its therapeutic use has received endorsement by multiple medical associations. We conducted a comprehensive overview of meta-analyses examining the efficacy of hypnosis to provide a foundational understanding of hypnosis in evidence-based healthcare, insight into the safety of hypnosis interventions, and identification of gaps in the current research literature.

**Methods:**

In our systematic review, meta-analyses of randomized controlled trials on the efficacy of hypnosis in patients with mental or somatic health problems compared to any control condition published after the year 2000 were included. A comprehensive literature search using Medline, Scopus, PsycINFO, The Cochrane Library, HTA Database, Web of Science and a manual search was conducted to identify eligible reviews. Methodological quality of the included meta-analyses was rated using the AMSTAR 2 tool. Effect estimates on various outcomes including at least three comparisons (*k* ≥ 3) were extracted and transformed into a common effect size metric (Cohen’s *d*). If available, information on the certainty of evidence for these outcomes (GRADE assessment) was obtained.

**Results:**

We included 49 meta-analyses with 261 distinct primary studies. Most robust evidence was reported for hypnosis in patients undergoing medical procedures (12 reviews, 79 distinct primary studies) and in patients with pain (4 reviews, 65 primary studies). There was a considerable overlap of the primary studies across the meta-analyses. Only nine meta-analyses were rated to have high methodological quality. Reported effect sizes comparing hypnosis against control conditions ranged from *d* = −0.04 to *d* = 2.72. Of the reported effects, 25.4% were medium (*d* ≥ 0.5), and 28.8% were large (*d* ≥ 0.8).

**Discussion:**

Our findings underline the potential of hypnosis to positively impact various mental and somatic treatment outcomes, with the largest effects found in patients experiencing pain, patients undergoing medical procedures, and in populations of children/adolescents. Future research should focus on the investigation of moderators of efficacy, on comparing hypnosis to established interventions, on the efficacy of hypnosis for children and adolescents, and on identifying patients who do not benefit from hypnosis.

**Clinical Trial Registration:**

https://www.crd.york.ac.uk/prospero/display_record.php?ID=CRD42023395514, identifier CRD42023395514

## 1 Introduction

A systematic review of reviews can provide an expanded view and broad examination of a body of information available for a certain topic (Hartling et al., 2012). Such reviews can highlight the evidence base for treatments with delineation of consistency, discrepancies, safety concerns, and efficacy (Aromataris et al., 2014; Faulkner et al., 2022). This type of generalized information is often used in the development of treatment guidelines and recommendations.

We conducted such an overview of reviews (also called “umbrella review”; Gates et al., 2022) focusing on meta-analyses that have been published in the last 20 years on the efficacy of hypnosis in various clinical fields. Our overview and the meta-analyses included herein are important for clinical hypnosis to be added to treatment guidelines and recommendations as an evidence-based approach. This type of achievement has recently been realized by clinical hypnosis as the North American Menopause Society (NAMS) recommends it with Level-I status (good and consistent scientific evidence), as a nonhormonal treatment to manage menopause-associated vasomotor symptoms (North American Menopause Society, 2023).

The documented use and investigation of hypnosis spans centuries and its therapeutic use has received endorsement by multiple medical associations (British Medical Association, 1955; Council on Mental Health, 1958; Palsson et al., 2023). According to APA Division 30 (Society of Psychological Hypnosis), *hypnosis* is defined as a “state of consciousness involving focused attention and reduced peripheral awareness characterized by an enhanced capacity for response to suggestion” (Elkins et al., 2015, p. 6). This definition was finalized after several iterations, due to the challenges of defining hypnosis when the mechanisms are complex and have been found in biological (e.g., functional connectivity, brain states), psychological (e.g., expectancy, hypnotizability), and social (e.g., rapport, demand characteristics) domains with no primary factor and variable combinations of factors across applications (Jensen et al., 2015). *Hypnotherapy* includes the therapeutic application of hypnosis, defined as the “use of hypnosis in the treatment of a medical or psychological disorder of concern” (Elkins et al., 2015, p. 7). The past several decades have yielded helpful research findings from investigations on the therapeutic use of hypnosis to treat a variety of somatic and mental concerns.

Over the past 20 years, the field of clinical hypnosis has seen an improvement in scientific rigor and new research has expanded to include randomized control trials and meta-analyses alike. A past systematic review of meta-analyses (Häuser et al., 2016) highlighted the safety and efficacy of hypnosis within medicine and found robust evidence for the use of hypnosis to reduce pain, emotional distress, duration of medical interventions, medication use, and symptoms related to irritable bowel syndrome. The authors indicate that helping patients learn and use self-hypnosis techniques can empower them to participate more in their treatment and enhance their autonomy. Hypnosis techniques were also identified to help build trust between patients and providers and many of these techniques do not require a formal hypnotic induction to be effective. Although some research has pointed out potential unwanted effects associated with hypnosis (Gruzelier, 2000), the authors’ conclusion regarding hypnosis as a safe intervention is consistent with a 2018 analysis of frequencies of adverse events in registered clinical trials which reported that there was zero report of a serious adverse event attributable to hypnosis and that the rate of “other adverse events” was 0.47% across 429 participants included in the studies (Bollinger, 2018). Further evidence of this is provided in a large meta-analysis on hypnosis for pain relief which included 3,632 patients across 85 trials concluding that hypnosis is both a safe and effective alternative to pharmaceutical approaches (Thompson et al., 2019).

A recent international survey that included nearly 700 hypnosis practitioners (Palsson et al., 2023) provides a general view of how hypnosis is utilized in clinical settings. Results from the survey revealed that hypnosis is most commonly used by clinical psychologists (42.7% of respondents reported this as their profession) and 60.5% of respondents reported offering hypnosis treatment in a private practice setting. Respondents were also asked to rate the effectiveness of specific applications of clinical hypnosis. Seven applications of hypnosis were rated as “highly effective” by at least 70% of respondents: stress reduction, enhancing well-being, preparing for surgery, anxiety, mindfulness, childbirth, and enhancing confidence. Conversely, the applications with the least amount of endorsement for being highly effective included obsessive-compulsive disorder, eating disorders, and weight loss. Almost two-thirds of respondents reported using video conferencing to provide hypnosis intervention and the majority of those professionals rated remote delivery to be as effective as in-person delivery. It is important to note that some have critiqued the survey in its effort to gather information about the “hypnosis styles” commonly used by respondents’ which had overlapping response options and unclear intention (McCann, 2023).

The recently published *Evidence-Based Practice in Clinical Hypnosis* (Milling, 2023) provides a helpful resource that outlines the evidence and use of hypnosis for issues such as depression, anxiety, pain, and smoking cessation. In the introductory chapter (Lynn and Green, 2023), the authors delineate what clinical hypnosis looks like in practice. More specifically, they describe that it usually begins with “prehypnotic information” characterized by inquiring about beliefs and previous experience with hypnosis, correcting misconceptions, discussing goals and potential suggestions, instilling positive expectancy, and answering any questions. This is typically followed by the hypnotic induction which conventionally includes instructive suggestions for eye fixation and closure, attention to breathing, calmness, and relaxation. After that, clinicians usually provide suggestions for “deepening” which focuses on intensifying and expanding the relaxation and feelings of calmness. This is traditionally followed by the hypnotic suggestions that deliberately aim to evoke a helpful emotional, psychological, and/or physiological experience based on the goals of treatment. Posthypnotic suggestions (those that elicit behavioral or mental activity after hypnosis) are often provided before re-alerting patients to normal consciousness.

A recent turning point in hypnosis research occurred in 2021 when the National Center for Complementary and Integrative Health (NCCIH) issued three funding opportunity announcements for mind-body intervention trials, and identified hypnotherapy as a treatment approach with “high programmatic priority” (National Center for Complementary and Integrative Health, 2021a,b,c). These funding mechanisms are consistent with the NCCIH strategic plan to pursue research that “[fosters] research on health promotion and restoration, resilience, disease prevention, and symptom management;” a top priority of the center (National Center for Complementary and Integrative Health, 2021d). Notably, these are the first grant opportunities issued by the NCCIH since 2015 to identify hypnotic interventions as a high priority research topic. The NCCIH recognizes evidence for the efficacy of hypnosis in the treatment of IBS, chronic pain, PTSD, and hot flashes (National Center for Complementary and Integrative Health, 2020). The NCCIH website also notes preliminary data for the use of hypnosis in smoking cessation and anxiety related to medical and dental procedures.

While there is extensive evidence regarding the efficacy of hypnosis for various mental and somatic concerns, its generalized efficacy is not clearly understood. We were interested in investigating the broad efficacy of hypnosis interventions on various problem-relevant outcomes compared to non-active or active control groups as reported in meta-analyses of randomized controlled trials which are considered to be at the top of the evidence hierarchy (Guyatt et al., 1995) and provide a foundational component of evidence-based healthcare. To our knowledge, this is the first overview of meta-analyses that summarizes relevant findings for clinical hypnosis. Hunt et al. (2018) suggested that overviews such as this can help filter the breadth of available information to improve decision making in healthcare and inform accurate development of treatment recommendations. We find this endeavor worthwhile to bring a greater awareness of hypnosis-specific interventions and to provide both researchers and clinicians a “user-friendly” overview of hypnosis research to more easily understand how clinical hypnosis can help, its overall safety, and in what areas more research is warranted.

## 2 Materials and methods

The reporting of this overview of reviews was guided by the standards of the Preferred Reporting Items for Overviews of Reviews (PRIOR) Statement (Gates et al., 2022).

### 2.1 Eligibility criteria

According to our a-priori review protocol (PROSPERO International prospective register of systematic reviews, registration number CRD42023395514), we considered the inclusion of meta-analyses synthesizing results from randomized controlled trials (RCTs) only, in addition to those reporting effects of RCTs in subgroup analyses. Eligible reviews included patients with mental or somatic health problems of any age demographic. Reviews on experimental studies were excluded. Meta-analyses needed to either focus explicitly on hypnosis or report effects of a hypnosis intervention in subgroup analyses. Any non-active or active control group was considered as eligible comparator. Analyses reporting pooled effect estimates had to be based on at least three comparisons (*k* ≥ 3). Inclusion was limited to reviews published after the year 2000 to ensure a higher chance of systematic and more rigorous meta-analytic methods, transparent and complete reporting of methods and results, and risk of bias judgement of the included studies (Moher et al., 1999).

### 2.2 Information sources and search strategy

A comprehensive literature search was conducted in MEDLINE, Scopus, PsycINFO, The Cochrane Library (Cochrane Database of Systematic Reviews, Cochrane Central Register of Controlled Trials (CENTRAL), Cochrane Methodology Register), Health Technology Assessment Database, and Web of Science (science and social science citation index). Within these databases, the search strategy included terms relating to or describing the intervention (hypnosis or hypnotherapy) and the study design (meta-analysis). There were no language restrictions, but an English abstract was required. The last search was conducted on 09.03.2023. The full MEDLINE search strategy is presented in Supplementary material 1. We adapted the search strategy used in MEDLINE to run properly in the other electronic databases. Further, we validated our search by verifying whether all trials reported in comprehensive yearly reviews of published meta-analyses and RCTs on the effectiveness of clinical hypnosis and hypnotherapy (published 2014–2021 in the German journal “Hypnose-ZHH” by Maria Hagl) were included. In addition, we conducted a manual search in the reference lists of the included reviews.

### 2.3 Selection process

Two authors (AH and JR) jointly decided whether a systematic review met the inclusion criteria of this overview of reviews. In case of redundant publications of the same study, only one publication was considered for inclusion and the overlap was noted.

### 2.4 Data collection process

Descriptive data of the reviews was extracted by one author (AH) who was previously trained. Initially, extracted information of a subset of five reviews was checked by another author (JR) who had extensive coding experience to calibrate data extraction and to ensure fidelity with the codebook.

Overlap in the included primary studies was identified by generating a studies-included-per-review matrix. Updates of existing reviews were marked as such. We did not exclude previous versions of updated meta-analyses to recognize scientific progress. If various publications of the same dataset were identified, only one publication was considered for inclusion, and we report this duplicate publication.

Outcome data was extracted by one author (JR) with extensive experience in meta-analytic effect size coding. Effect sizes were extracted per comparison and outcome, regardless of primary study overlap across the reviews. We used standardized mean differences (Cohen’s *d*) with 95% confidence interval to display differences between hypnosis and control conditions, applying the interpretation guideline of Cohen (1992), regarding 0.20, 0.50, and 0.80 as small, medium, and large effect sizes, respectively. Positive effect sizes indicate a superiority of the hypnosis condition, while negative effect sizes suggest a superiority of the comparison condition. If outcome data were reported in different effect size formats (e.g., odds ratio), we transformed it into Cohen’s *d* by using Comprehensive Meta-Analysis (Biostat) software.

### 2.5 Data items

We extracted the following data items from the included reviews: descriptive characteristics of the meta-analyses (and their included primary studies), data to inform risk of bias assessment of the meta-analyses (and their included primary studies), quantitative outcome data, and certainty of evidence for important outcomes [e.g., heterogeneity of results, Grading of Recommendations, Assessment, Development and Evaluation (GRADE) assessments; Guyatt et al., 2008]. Data selection and coding were realized by two independent raters (AH and JR). Disagreements were resolved by consensus discussion. When necessary, a third researcher (CA) was consulted. If information on an aggregate level was missing in the included reviews, we checked the descriptions of the primary studies as reported in the reviews.

### 2.6 Risk of bias assessment

Methodological quality of the included meta-analyses was appraised and rated using the second edition of A MeaSurement Tool to Assess systematic Reviews (AMSTAR 2; Shea et al., 2017). The AMSTAR 2 is comprised of 16 domains (seven critical and nine noncritical) that allow for rating beyond a dichotomous “yes” or “no” to provide one of four overall ratings of confidence in the results of the review: high, moderate, low, and critically low. The critical domains included inquiries on protocol preregistration, adequacy of literature search, justification for exclusions, risk of bias, meta-analytic methods, interpretation of results, and publication bias. Meta-analyses in the present study were first rated on the seven critical domains because the presence of one or more critical flaw would result in an unchangeable rating of low (one critical flaw with or without non-critical weaknesses) or critically low (more than one critical flaw with or without non-critical weaknesses), respectively. Meta-analyses that did not have any critical flaws were rated on the other nine non-critical domains to earn an overall rating of moderate (more than one non-critical weakness) or high (no or one non-critical weakness), respectively.

### 2.7 Synthesis methods

Results were summarized graphically using a common effect size metric (standardized mean difference, Cohen’s *d*). We did not synthesize the results of the reviews because of the diversity of populations, comparators, and outcomes and the considerable overlap of the primary studies across the included reviews.

### 2.8 Reporting bias assessment

Reporting bias considered in the included reviews (i.e., potential publication bias in the review results) was assessed using item 15 of the AMSTAR 2 tool (Shea et al., 2017). Two independent raters (CA and JR) assessed whether the authors of the included meta-analyses carried out an adequate investigation of publication bias (small study bias) and discuss its likely impact on the results of the review. Disagreements were resolved by consensus discussion.

### 2.9 Certainty assessment

For each comparison and outcome, we extracted information on the certainty for the body of evidence [i.e., GRADE assessments (Guyatt et al., 2008)] if it was reported in the included reviews. We also collected specific information on the inconsistency of results, which is commonly reported by indicators of heterogeneity (Higgins et al., 2003).

## 3 Results

### 3.1 Systematic review selection

**Figure 1:**
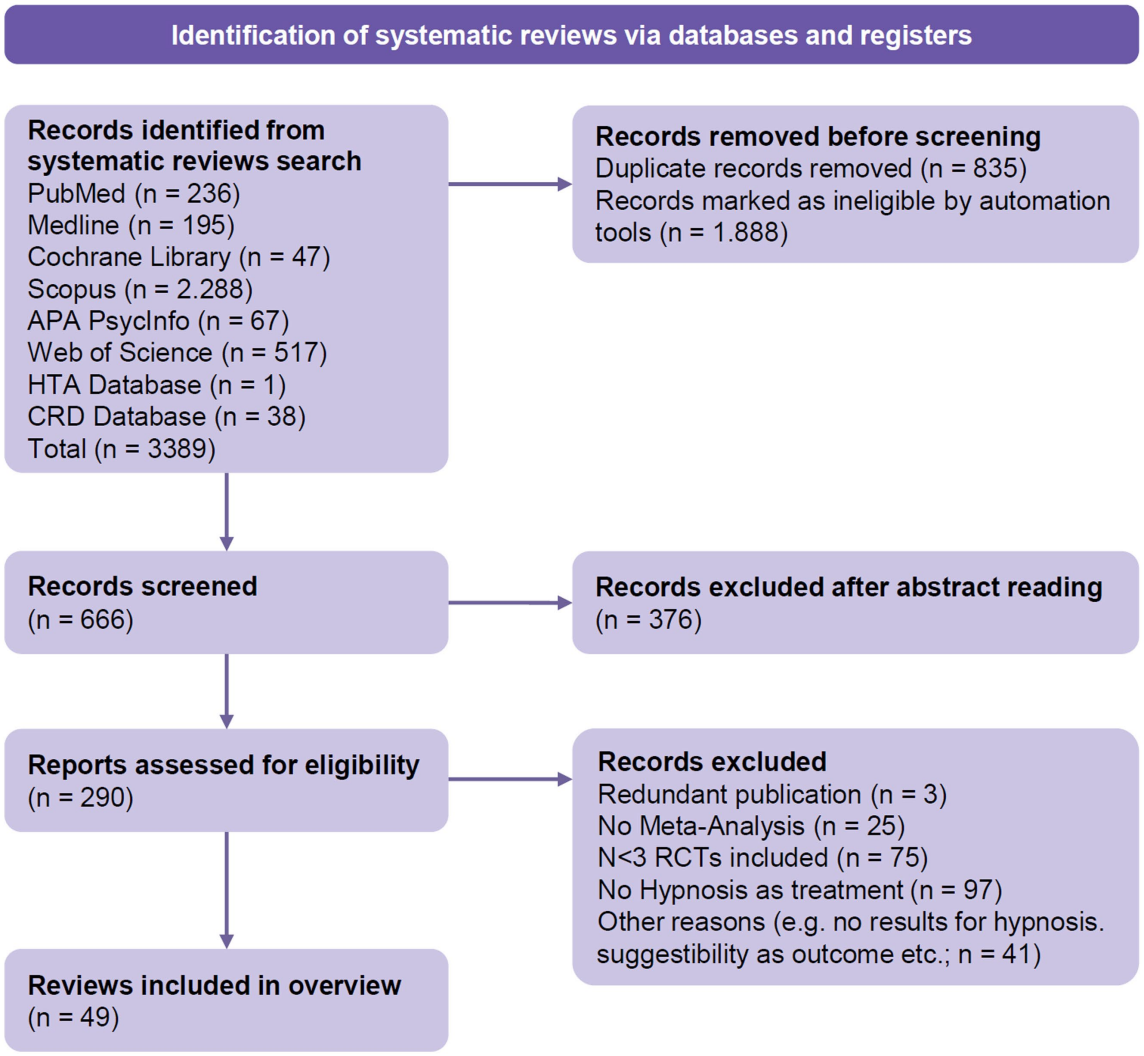
PRIOR flow diagram (Gates et al., 2022).

Our comprehensive search in various relevant data bases resulted in a total of 3,389 records. Of these, 2,723 duplicate and ineligible records were excluded. We screened 666 records, and after exclusion during abstract screening, 290 records remained and were screened for eligibility via full-text inspection. Of these, 241 reports were excluded due for reasons such as duplicate publication or failure to meet the inclusion criteria. Finally, 49 reviews were included in the present synthesis (Figure 1; Supplementary material 2). Studies that appeared to meet the inclusion criteria but were excluded during the selection process are presented in Supplementary material 3.

### 3.2 Characteristics of the systematic reviews

The 49 included meta-analyses were published between 2003 and 2022 and comprised 261 primary studies (published between 1962 and 2021). Twelve reviews (including 79 distinct primary studies) focused on medical procedures (e.g., surgery, needle-related procedures, etc.), five reviews were on labor and childbirth (10 primary studies), four reviews examined hypnosis for patients with pain (65 primary studies), and another five reviews focused on cancer (26 primary studies). We also identified 10 reviews on irritable bowel syndrome (19 primary studies), one review on obesity (16 studies), four reviews on smoking cessation (14 studies), and five were on patients with symptoms of mental/psychosomatic disorders (including insomnia; 37 primary studies). Additionally, we included three comprehensive reviews (112 primary studies) on various disorders. These reviews reported effects pooled across all studies and outcomes, but also yielded subgroup data for specific problems. Characteristics of the included meta-analyses are presented in Table 1 (more details are provided in Supplementary Table S1). The number of included hypnosis trials per review ranged from three (Smith et al., 2003; O’Toole et al., 2016) to 57 (Flammer and Bongartz, 2003). The number of patients included in the primary studies of the reviews ranged from 94 (O’Toole et al., 2016) to 4,269 (Holler et al., 2021), with a median of 502 (interquartile range 253 to 1,409). The majority of the reviews (*n* = 32, 65.3%) included adults only. In four reviews (8.1%), only children and/or adolescents were considered, 11 reviews (22.4%) included patients of all ages, and two reviews did not report information on the age of the study population. Of the 49 reviews, 18 (36.7%) included RCTs on hypnosis only, 23 (46.9%) focused on RCTs on different interventions and reported subgroup results for hypnosis, and eight reviews (16.3%) included randomized and non-randomized studies on hypnosis and reported subgroup results for RCTs. Seventeen reviews provided information on the assessment of hypnotizability within the primary studies. In eight of these reviews, results on the relationship between hypnotizability and outcome were reported (more or less detailed; Supplementary Table S1). Hypnosis was compared against various non-active and active control groups, predominantly against treatment as usual control groups (36 meta-analyses, 73.5%), attention control/placebo (31, 63.3%), active control/alternative treatment (17, 34.7%), waitlist control conditions (16, 32.7%), and no treatment control groups (13, 26.5%, see Table 1; Supplementary Table S1).

**Table 1:** Characteristics of the included systematic reviews.

Review	No. of included studies (participants)	Date of last search	Type of included studies	Population (age; % female)	Intervention	Comparison condition	Reported outcomes with *k* ≥ 3
**Medical procedures**							
Holler et al. (2021)	50 (4,269)	January 2021	RCTs on hypnosis only	Adults (mean age: 51 yrs; 66%)	Hypnosis as an adjunct to surgical standard care, implemented pre-, intra- and/or postoperatively, provided face-to-face or as a pre-recorded tape/CD; Intervention time: 15–60 min	(1) TAU (2) AC	Mental distress; Pain Medication consumption Recovery; Physiological parameters; Procedure time
Tefikow et al. (2013)	34 (2,597)	September 2011	RCTs on hypnosis only	Adults (mean age: 40 yrs; 60%)	Hypnosis as an adjunct to surgical standard care, implemented pre-, intra- and/or postoperatively, provided face-to-face or as a pre-recorded tape/CD; Intervention time: 3–240 min	(1) TAU (2) AC	Emotional distress; Pain Medication consumption Recovery; Physiological parameters; Procedure time
Kekecs et al. (2014)	13 (1,028)	February 2014	RCTs on hypnosis only	Adults (NR)	Use of hypnosis and therapeutic suggestions to alleviate surgical side effects, implemented pre-, during- and after-surgery, live and recorded	(1) TAU (2) AC	Anxiety; Pain intensity Pain medication Nausea
Schnur et al. (2008)	19 (1,723)	February 2008	RCTs on hypnosis only	Children and adults (NR)	Hypnosis for reducing emotional stress during procedure, implemented pre-, during- and after-surgery, only live	(1) TAU (2) AC	Mental distress
Noergaard et al. (2019)	7 (1,231)	July 2018	RCTs on hypnosis only	Adults (18–94 yrs; 71%)	Hypnotic analgesia in the management of procedural pain in minimally invasive procedures, implemented pre- and during the procedure, face-to-face and via recording; Intervention time: 16–195 min	(1) TAU + intravenous analgesia (2) TAU (without pain medication)	Length of procedure Adverse events
Zeng et al. (2022)	8 (1,242)	January 2022	RCTs on hypnosis only	Adults (18–92 yrs; 100%)	Hypnosis before general anesthesia on patients undergoing minor surgery for breast cancer and self-hypnotic relaxation exercise; Intervention time: 2–20 min	TAU	Postoperative pain Postoperative anxiety Procedure time Postoperative nausea and vomiting
Burghardt et al. (2018)	5 (255)	August 2017	RCTs on different interventions including hypnosis	Adults (NR)	Listening to recorded hypnosis during procedure (partly with relaxation music, suggestions for bleeding and edema control) Intervention time: 20–66 min	(1) TAU (2) AC	Mental distress
Uman et al. (2006)	5 (196)	February 2005	RCTs on different interventions including hypnosis	Children and adolescents (3–16 yrs; NR)	Training in hypnosis and self-hypnosis; Hypnotic suggestion using the child’s favourite story; Visual imagery and analgesic suggestions, relaxation techniques	(1) TAU (2) AC (3) Non-directed play	Self-reported pain Self-reported distress Behavioral measures of distress
Uman et al. (2013)-update of Uman et al. (2006)	7 (255)	March 2013	RCTs on different interventions including hypnosis	Children and adolescents (3–16 yrs; NR)	Training in hypnosis and self-hypnosis; Hypnotic suggestion using the child’s favourite story; Visual imagery and analgesic suggestions, relaxation techniques; Ericksonian hypnosis via hypnotherapist	(1) TAU (2) AC (3) Non-directed play	Self-reported pain Self-reported distress Behavioral measures of distress
Birnie et al. (2018)-update of Uman et al. (2013)	8 (295)	September 2017	RCTs on different interventions including hypnosis	Children and adolescents (3–16 yrs; 60%)	Training in hypnosis and self-hypnosis; Hypnotic suggestion using the child’s favourite story; Visual imagery and analgesic suggestions, relaxation techniques; Ericksonian hypnosis via hypnotherapist; Hypnosis intervention via headphones	(1) TAU (2) AC (3) Non-directed play (4) Noise-cancelling headphones	Self-reported pain Self-reported distress Behavioral measures of distress
Provençal et al. (2018)	6 (234)	August 2014	RCTs on hypnosis only	Adults (16–65 yrs; NR)	Hypnosis via Barber’s rapid induced analgesia modified for wound debridement, with posthypnotic cues for comfort; Muscle relaxation and pleasant memory; Intervention time: 15–25 min	(1) TAU (2) AC (3) No treatment	Pain
Scheffler et al. (2018)	6 (213)	May 2016	RCTs on different interventions including hypnosis	Adults (NR)	Hypnosis via Barber’s rapid induced analgesia modified for wound debridement, with posthypnotic cues for comfort and relaxation	(1) TAU (2) AC	Pain
**Labor and childbirth**							
Madden et al. (2012)	7 (1,213)	January 2012	RCTs on different interventions including hypnosis	Adults (NR; 100%)	Antenatal hypnosis training in groups/individual; Hypnosis during labor; + audio-recording for home practice Intervention time: 20 min	(1) TAU (2) AC	Use of pharmacological pain relief/anesthesia Spontaneous vaginal birth; Assisted vaginal birth; Ceasarean section Use of epidural/neuroaxial block
Madden et al. (2016)-update of Madden et al. (2012)	9 (1,741)	September 2015	RCTs on different interventions including hypnosis	Adults (NR; 100%)	Antenatal hypnosis training in groups/individual; Hypnosis during labor; + audio-recording for home-practice Intervention time: 20–90 min	(1) TAU (2) AC	Use of pharmacological pain relief/anesthesia Spontaneous vaginal birth; Assisted vaginal birth; Ceasarean section Use of epidural/neuroaxial block Induction of labor Augmentation of labor
Cyna et al. (2004)	3 (142)	March 2004	Studies on hypnosis including RCTs	Adults (NR; 100%)	Individual hypnosis (standard script used in labor, including relaxation and focused attention)	(1) TAU (2) Placebo (3) Active control	Use of pharmacological pain relief
Smith et al. (2003)	3 (167)	July 2002	RCTs on different interventions including hypnosis	Adolescents and adults (18 or younger-35 yrs; 100%)	Individual hypnosis with guided imagery; Self-hypnosis in groups Intervention time: 60 min	(1) TAU (2) Active control	Use of pharmacological pain relief
Smith et al. (2006)-update of Smith et al. (2003)	5 (727)	February 2006	RCTs on different interventions including hypnosis	Adolescents and adults (18 or younger-35 yrs.; 100%)	Individual hypnosis with guided imagery; Self-hypnosis in groups Intervention time: 60 min	(1) TAU (2) Active control	Use of pharmacological analgesia; Spontaneous vaginal birth; Augmentation with oxytocin
**Pain**							
Langlois et al. (2022)	9 (530)	May 2021	RCTs on hypnosis only	Adults (34–81 yrs; NR)	Hypnosis via hypnotherapist, afterwards self-hypnosis via audiotape; Intervention time: 30–90 min	(1) TAU (2) AC (3) No treatment (4) Active control	Pain intensity post-treatment/follow-up Pain interference with daily activities
Garland et al. (2020)	12 (932)	March 2018	RCTs on different interventions including hypnosis	Adults (NR)	Hypnosis partly in person, partly via recording	(1) TAU (2) AC (3) Active control	Pain
Milling et al. (2021)	32 (1,409)	April 2019	Studies on hypnosis including RCTs	NR	Not specified	(1) TAU (2) AC (3) Waitlist (4) No treatment	Pain
Zech et al. (2017)	3 (134)	February 2016	RCTs on different interventions including hypnosis	Adults (NR)	Traditional and Ericksonian hypnosis, partly combined with CBT Intervention time: 60–120 min	(1) TAU (2) AC (3) Waitlist (4) Active control	Sleep problems
**Cancer**							
Richardson et al. (2007)	4 (149)	March 2005	RCTs on hypnosis only	Children, adolescents and adults (5–49 yrs; NR)	Individual hypnosis with instructions for self-hypnosis; Individualized imaginative fantasy with suggestions; Relaxation, imagery and tailored hypnosis with suggestions + instruction for home practice Intervention time: 30–90 min	(1) TAU (2) Therapist contact	Nausea and vomiting
Danon et al. (2022)	3 (130)	May 2020	RCTs on different interventions including hypnosis	Adults (mean age: 55.9 yrs; 96%)	Hypnosis and supportive-expressive therapy + education in groups; Instructions for self-hypnosis + pharmacological treatment Intervention time: 60–90 min Valencia model of waking hypnosis with CBT	(1) TAU (2) Waitlist (3) No treatment (4) Active control	Pain
Jong et al. (2020)	4 (206)	March 2016	RCTs on different interventions including hypnosis	Children and adolescents (3–16 yrs; NR)	Hypnotic induction, active imagery, tailored, deep muscle relaxation, and analgesic suggestions, directed by therapist + self-hypnosis training	(1) TAU (2) AC	Self-reported pain
Nunns et al. (2018)	6 (287)	July 2017	RCTs on different interventions including hypnosis	Children, adolescents and adults (NR)	Hypnosis + CBT; Direct and indirect suggestions; Imaginative involvement; Intervention time: 15–40 min	(1) TAU (2) AC	Anxiety Pain
Chen et al. (2017)	13 (321)	May 2015	Studies on hypnosis including RCTs	Children, adolescents and adults (5–87 yrs; NR)	Individual hypnosis with therapist, additional instructions for self-hypnosis with audiotape Intervention time: 20–50 min	(1) TAU (2) AC (3) Active control	Anxiety
**Irritable bowel syndrome**							
Ford et al. (2019)	5 (278)	December 2013	RCTs on different interventions including hypnosis	Adults (NR)	Gut-directed hypnotherapy, partly with audiotapes; Intervention time: 30–60 min	(1) TAU (2) Symptom monitoring (3) Supportive therapy (4) Placebo	Response to therapy (global IBS symptoms or abdominal pain)
Black et al. (2020)-update of Ford et al. (2019)	6 (739)	January 2020	RCTs on different interventions including hypnosis	Adults (NR)	Gut-directed hypnotherapy (individual vs. in groups); Intervention time: 30–60 min	Waitlist	IBS symptoms
Krouwel et al. (2021)	7 (723)	April 2020	RCTs on hypnosis only	Adults (18–65 yrs; 63.3–86.3%)	Gut-directed hypnotherapy, individual and in groups; Integrative therapy (including psychodynamics, GDH and education); Intervention time: 45–90 min	(1) TAU (2) Placebo (3) No treatment (4) Alternative treatment	IBS symptoms
Lee et al. (2014)	7 (374)	January 2013	RCTs on hypnosis only	Adults (18–70 yrs; 80%)	Gut-directed hypnotherapy Intervention time: 30–60 min	(1) Any other conventional treatment (2) No treatment	Abdominal pain
Schaefert et al. (2014)	8 (464)	June 2013	RCTs on hypnosis only	Adults (NR)	Manchester approach Gut-directed hypnotherapy (Partly individual and in groups) Intervention time: 30–60 min	(1) TAU (2) AC (3) Waitlist (4) No treatment (5) Active control	Adequate symptom relief; Global gastrointestinal score; Pain; Diarrhea; constipation; Bloating/distension; Health-related quality of life; Depression; Anxiety
Henrich et al. (2015)	5 (255)	May 2013	RCTs on different interventions including hypnosis	Adults (NR)	Gut-directed hypnotherapy Intervention time: 30–60 min	(1) TAU (2) Waitlist (3) Symptom monitoring	Pain; Composite symptoms; Bowel dysfunction; Psychological distress Health-related quality of life
Laird et al. (2016)	5 (253)	August 2015	RCTs on different interventions including hypnosis	Adults (NR)	Hypnotherapy, individual and in groups	Active and non-active controls	Gastrointestinal symptoms
Laird et al. (2017)	4 (223)	August 2015	RCTs on different interventions including hypnosis	Adults (NR)	Hypnotherapy, individual and in groups	(1) TAU (2) Waitlist (3) Active control	Daily functioning
Peng et al. (2021)	11 (509)	September 2020	RCTs on different interventions including hypnosis	Adults (NR)	Individual gut-directed hypnotherapy; GDH + supportive talks; General hypnotherapy; Group hypnotherapy	Supportive treatments	Various outcomes
Shah et al. (2020)	6 (NA)	NR	RCTs on different interventions including hypnosis	Adults (NR)	NA	(1) TAU (2) Waitlist (3) Active control	Gastrointestinal symptoms
**Obesity**							
Milling et al. (2018)	(A) 14 (882) (B) 11 (573)	December 2016	Studies on hypnosis including RCTs	Adolescents and adults (NR)	Hypnosis + training in self-hypnosis; Hypnosis alone; Hypnosis + CBT	(1) TAU (2) AC (3) No treatment (4) Hypnosis + CBT vs. CBT	Weight loss at post treatment and at follow-up
**Smoking cessation**							
Barnes et al. (2010)	11 (1,221)	July 2010	RCTs on hypnosis only	Adults (30–40 yrs; NR)	Hypnosis alone (individual or in groups); Hypnosis + audiotape for home-practice; Intervention time: 30–150 min	(1) Brief attention/cessation advice (2) Psychological treatment	Smoking cessation at 6+ months follow-up
Barnes et al. (2019)-update of Barnes et al. (2010)	14 (1,926)	July 2018	RCTs on hypnosis only	Adults (30–40 yrs; NR)	Hypnosis alone (individual or in groups); Hypnosis + audiotape for home-practice; Intervention time: 30–150 min	Attention-matched behavioural treatments	Smoking cessation at 6+ months follow-up
Tahiri et al. (2012)	4 (273)	December 2010	RCTs on different interventions including hypnosis	Adults (mean age 35.6–42.7 yrs; 48.7–70%)	Hypnosis by hypnotherapist, family physician or psychologist; Intervention time: 40–150 min	Waitlist	Abstinence
Hartmann-Boyce et al. (2021)	14 (1,926)	July 2020	RCTs on different interventions including hypnosis	Adults (30–40 yrs; NR)	Hypnosis alone (individual or in groups); Hypnosis + audiotape for home-practice; Intervention time: 30–150 min	(1) No smoking cessation support (2) Waitlist (3) AC	Smoking cessation at 6+ months follow-up
**Symptoms of mental/psychosomatic disorders**							
Shih et al. (2009)	6 (258)	NR	RCTs on hypnosis only	Adults (18–81 yrs; 67.5%)	Hypnosis alone (individual or in groups); Hypnosis + audiotape for home-practice; Intervention time: 30–60 min	(1) TAU (2) No treatment	Depressive symptoms
O’Toole et al. (2016)	3 (94)	NR	Studies on hypnosis including RCTs	Adolescents and adults (NR; 0–79%)	Hypnotherapy and “spiritual hypnosis-assisted therapy”	(1) No treatment (2) Waitlist (3) Pharmacotherapy	PTSD symptom reduction
Rotaru and Rusu (2016)	4 (144)	January 2014	Studies on hypnosis including RCTs	Children, adolescents and adults (9.38–42 yrs; 0–88%)	Symptom-orientated hypnotherapy; Manualized abreactive ego therapy Intervention time: 90 min	(1) No treatment (2) Waitlist (3) Pharmacotherapy (4) Placebo	PTSD symptom reduction at post-treatment and at 4 weeks follow-up
Flammer and Alladin (2007)	21 (843)	NR	RCTs on hypnosis only	Children, adolescents and adults (NR)	Classical hypnosis; Modern hypnosis; Mixed form of hypnosis, Ericksonian hypnosis	(1) Waitlist (2) No treatment	Pooled across all reported outcomes
Lam et al. (2015)	3 (75)	March 2014	RCTs on hypnosis only	Adults (mean age: 45.2 yrs; 52.8%)	Hypnosis (face-to-face; Delivered via internet); Intervention time: 20–90 min	(1) Placebo (2) Waitlist	Sleep onset latency
**Various disorders**							
Ramondo et al. (2021)	39 (1,500)	November 2018	Studies on hypnosis including RCTs	Children, adolescents and adults (NR)	Hypnosis + CBT	CBT alone	Primary study outcome at post-treatment and at follow-up
Flammer and Bongartz (2003)	57 (2,411)	2002	Studies on hypnosis including RCTs	Children, adolescents and adults (NR)	Classical hypnosis (direct suggestions for relaxation, imagination and for alleviation of symptoms); Modern hypnosis (indirect suggestions for relaxation, imagination or for symptom reduction, application of metaphors, symbolizations)	Waitlist	Various outcomes
Eason and Parris (2019)	17 (4,176)	NR	RCTs on hypnosis only	Children, adolescents and adults (NR)	Hypnosis alone (individual or in groups); Hypnosis + audiotape for home-practice; Intervention time: 50–90 min	(1) TAU (2) Waitlist (3) Other treatment	Various outcomes

AC, attention control; CBT, cognitive-behavioral therapy; GDH, gut-directed hypnotherapy; IBS, irritable bowel syndrome; NR, not reported; RCT, randomized controlled trial; TAU, treatment as usual; yrs, years.

### 3.3 Primary study overlap

There was a considerable overlap of primary studies across the included systematic reviews (Supplementary Figure S1). Each primary study was included in *M* = 2.21 (SD = 1.77) reviews. Of the 261 distinct primary studies, 129 (49.4%) were considered in only one review, while some studies were included in several reviews: Lindfors et al. (2012) was included in 10 reviews; Moser et al. (2013) and Liossi et al. (2006) were included in 9 reviews, Katz et al. (1987), Lang et al. (2000) and Liossi and Hatira (2003) were each included in 8 reviews (Supplementary Table S2).

### 3.4 Risk of bias in systematic reviews

**Figure 2:**
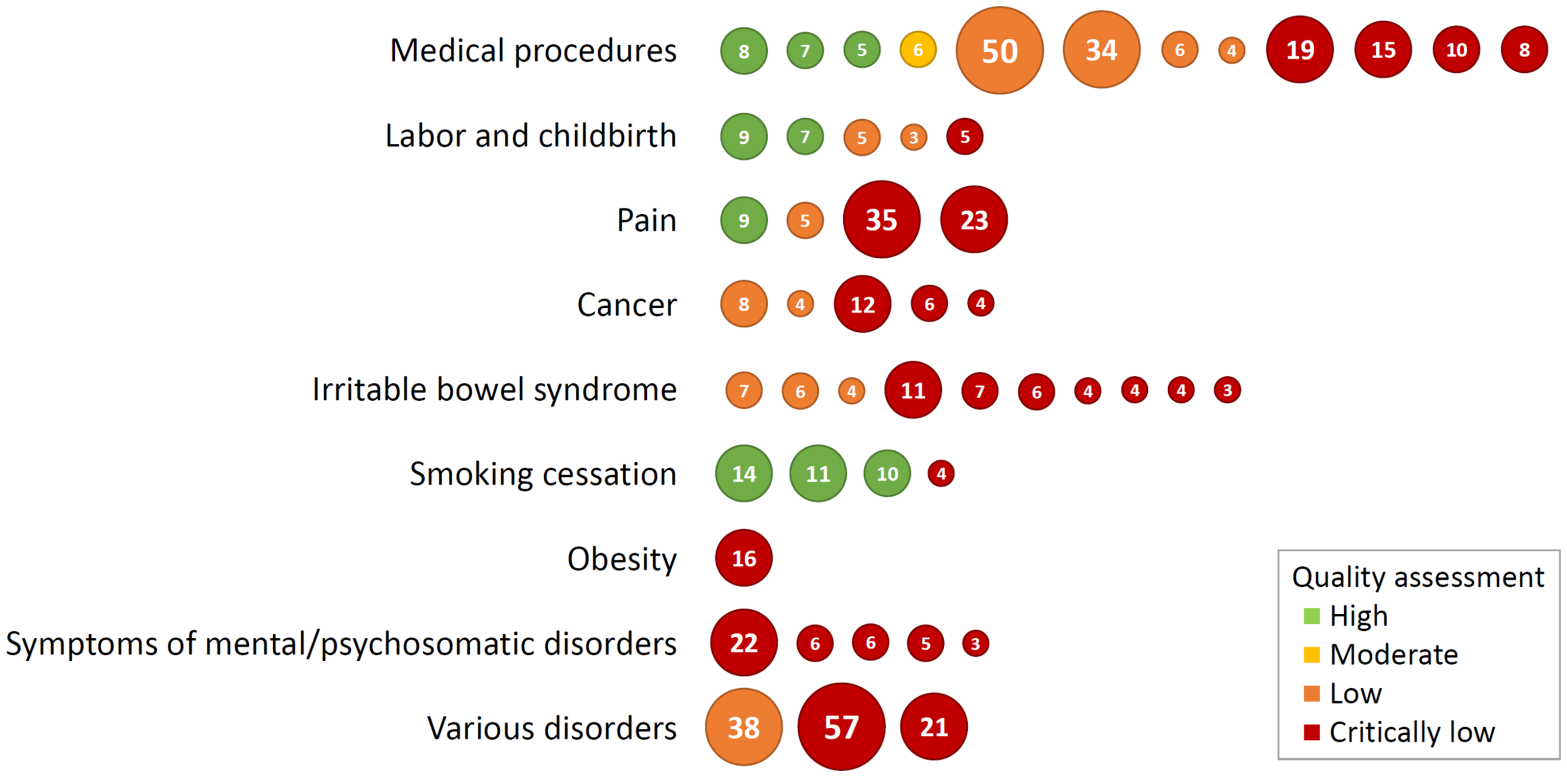
Quality assessment of the included reviews showing overall ratings of the AMSTAR 2 tool. Each circle represents one included review, and the number in circles are studies included in each review. Order of circles is from high quality (left) to low quality (right).

The assessment of the methodological quality of the included reviews using the AMSTAR 2 tool (Shea et al., 2017) revealed only a few overall quality ratings of *high*. The methodological quality of nine reviews (18.4%, including three of four reviews on smoking cessation) was judged as high, indicating no weaknesses in critical domains. One review was assessed as having *moderate* overall quality due to two non-critical weaknesses and *low* overall quality was judged for 13 reviews (26.5%), indicating one critical flaw with or without non-critical weaknesses. Of these low quality reviews, nine did not provide a list of excluded studies and justified the exclusions, three did not assess the presence of a publication bias and discuss its likely impact on the results, and one review did not register an *a-priori* review protocol. A majority of 26 reviews (53.1%) had a methodological quality judged as *critically low* (i.e., having more one critical flaw with or without non-critical weaknesses). Figure 2 shows a summary of the quality assessment of the included reviews. Results of the AMSTAR 2 assessment on the critical items and an overall rating for the included studies are shown in Supplementary Table S3.

### 3.5 Summary of results

**Figure 3:**
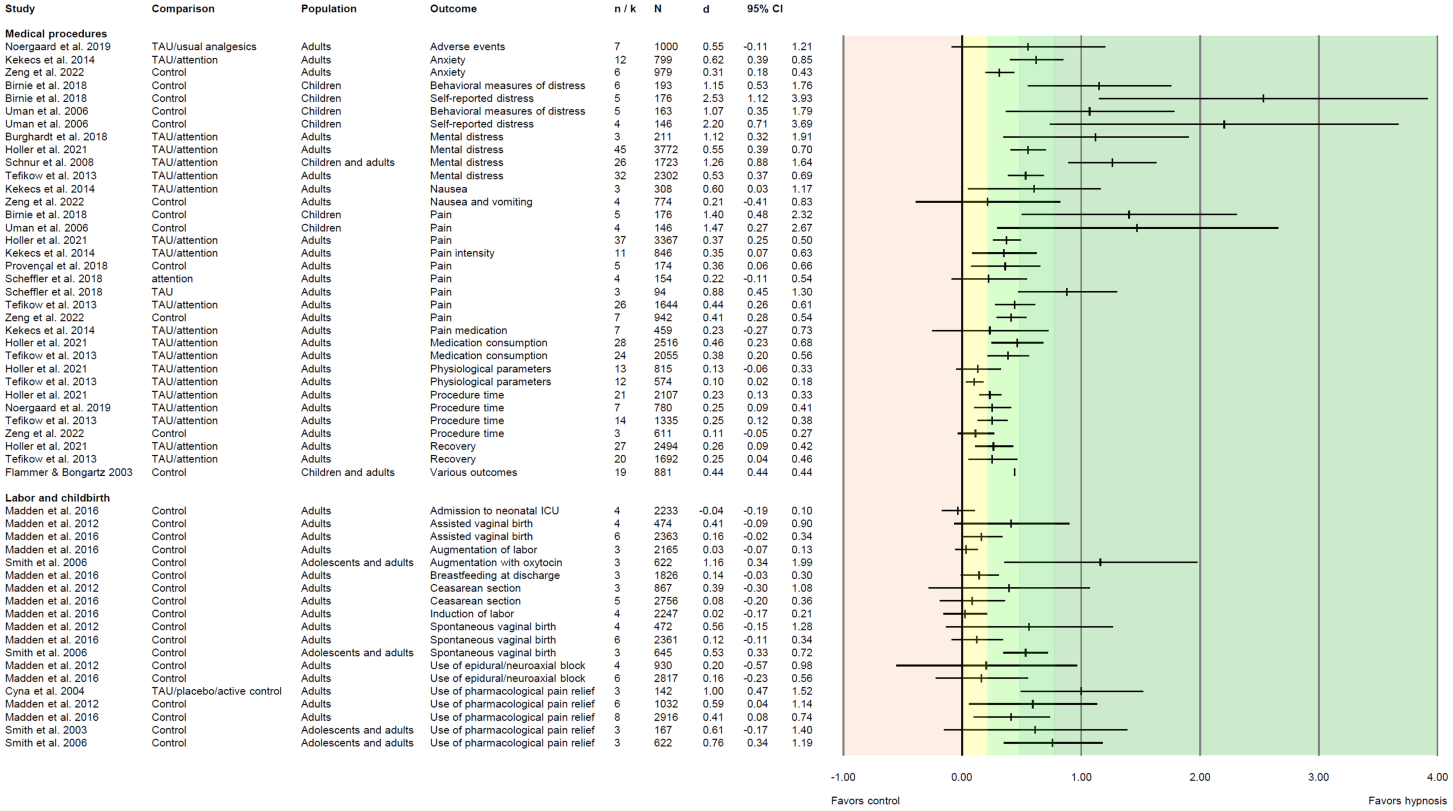
Effect sizes for comparing hypnosis against control conditions in patients undergoing medical procedures or labor/childbirth. Colored areas indicate different effect size interpretation as negative (red), very small/*d* < 0.2 (yellow), and small/*d* ≥ 0.2, medium/*d* ≥ 0.5, or large effects/*d* ≥ 0.8 (green). Attention, attention control; ICU, intensive care unit; TAU, treatment as usual.

**Figure 4:**
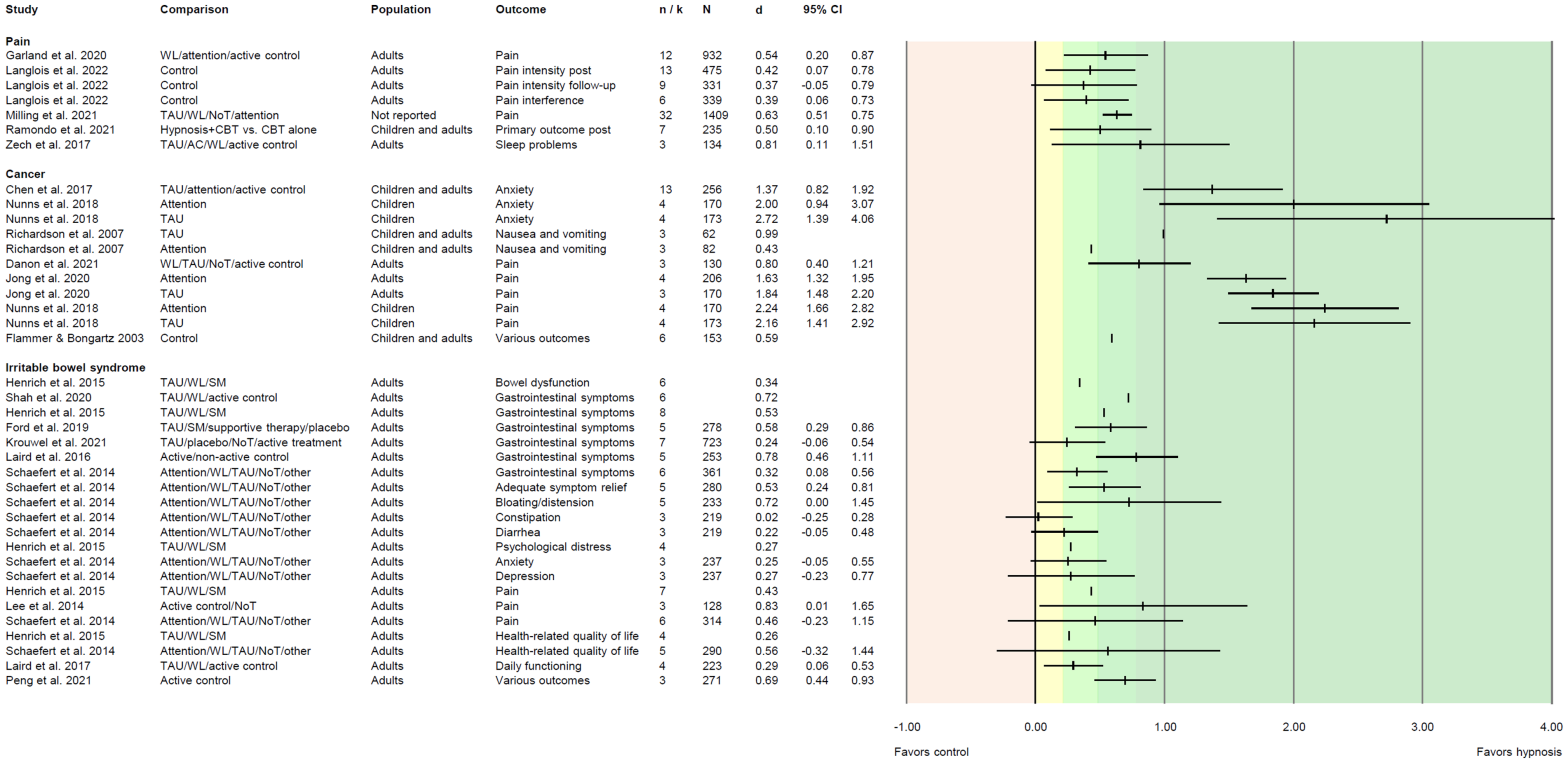
Effect sizes for comparing hypnosis against control conditions in patients with pain, cancer, or irritable bowel syndrome. Colored areas indicate different effect size interpretation as negative (red), very small/*d* < 0.2 (yellow), and small/*d* ≥ 0.2, medium/*d* ≥ 0.5, or large effects/*d* ≥ 0.8 (green). Blank cells indicate missing information. Attention, attention control; CBT, cognitive-behavioral therapy; NoT, no treatment control; SM, symptom monitoring; TAU, treatment as usual; WL, waitlist control.

**Figure 5:**
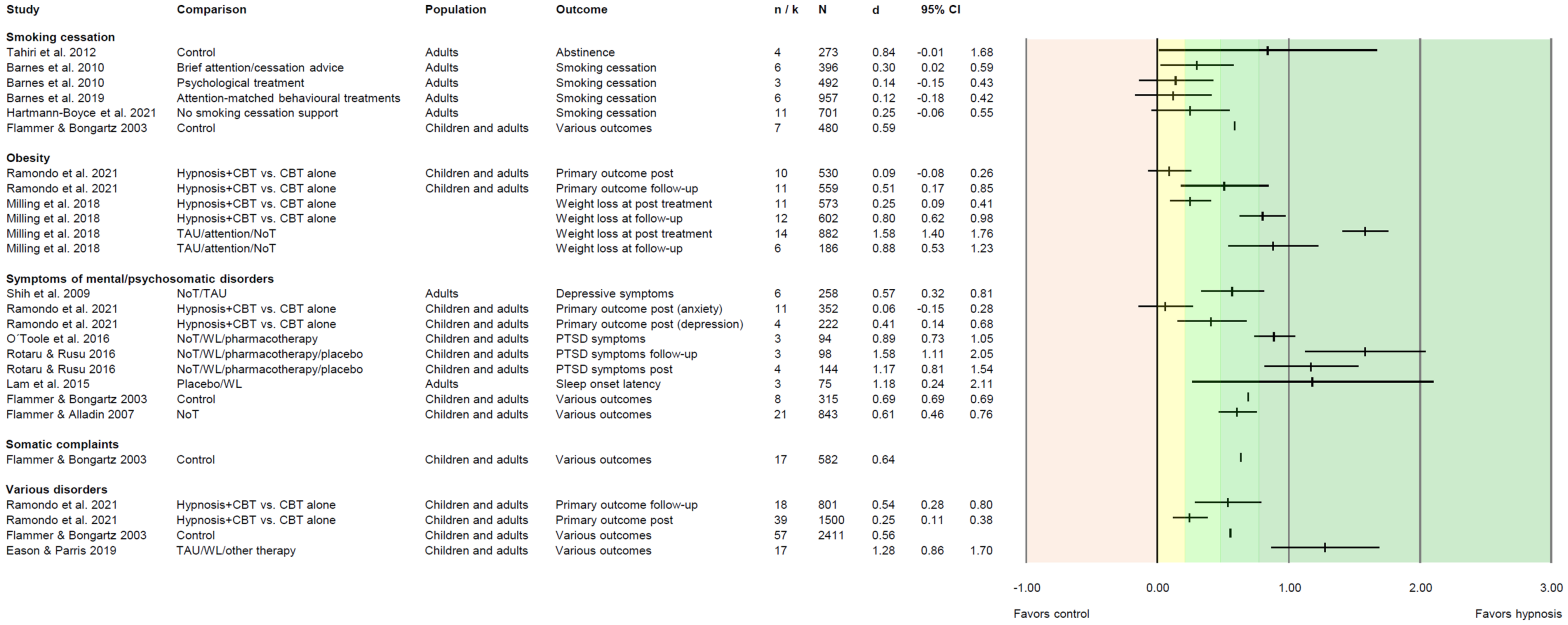
Effect sizes for comparing hypnosis against control conditions for smoking cessation, obesity, patients with psychological/psychosomatic symptoms or somatic complaints, and for various disorders. Colored areas indicate different effect size interpretation as negative (red), very small/*d* < 0.2 (yellow), and small/*d* ≥ 0.2, medium/*d* ≥ 0.5, or large effects/*d* ≥ 0.8 (green). Blank cells indicate missing information. Attention, attention control; CBT, cognitive-behavioral therapy; NoT, no treatment control; TAU, treatment as usual; WL, waitlist control.

We extracted *i* = 118 effect sizes from the included reviews. Effect sizes (Cohen’s *d*) were based on *M* = 9.19 primary studies including *M* = 796 patients, and ranged from *d* = −0.04 to *d* = 2.72 (Figures 3–5). More than half of the effects (*i* = 75, 63.6%) were reported as significant. According to Cohen (1992), about one third of the effects (*i* = 41, 34.7%) can be regarded as small, 25.4% (*i* = 30) as medium, and 28.8% (*i* = 34) as large.

#### 3.5.1 Medical procedures

Reviews on patients undergoing medical procedures made up the largest number of our synthesis (*n* = 12). In these reviews, 34 effect sizes were reported ranging from *d* = 0.1 to *d* = 2.53, 28 effect sizes were significant, 17 can be interpreted as small, five as medium, and nine as large effects. The largest effects appeared in populations of children with needle-related pain and distress (Uman et al., 2006; Birnie et al., 2018), with effect sizes between *d* = 1.07 and *d* = 2.53. Homogeneously across all reviews, (very) small effects were seen for physiological parameters, procedure time, and recovery. Medium to large effects appeared for mental distress including anxiety, except for one review (Zeng et al., 2022) that reported a small effect (Figure 3).

#### 3.5.2 Labor and childbirth

Five reviews examined hypnosis in labor and childbirth, reporting 19 effect sizes ranging from *d* = −0.04 to *d* = 1.16. Eighteen effect sizes were positive, but only four proved to be significant. Among the reported effects, four were small, five were medium, and another five were large. For the outcome “use of pharmacological pain relief,” the largest effects appeared (five effect sizes; all but one medium to large and significant). The majority of effects on other birth-related outcomes were (very) small and not significant (Figure 3).

#### 3.5.3 Pain

Effects on hypnosis in patients with various types of clinical pain including fibromyalgia were examined in five reviews (one reported effects within a subgroup analysis; Ramondo et al., 2021). Seven positive effect sizes (six significant, three each small or medium, one large) were reported, ranging from *d* = 0.37 to *d* = 0.81 (Figure 4). Six effects were reported for pain (intensity), and one review reported results for the reduction of sleep problems (*d* = 0.81; Zech et al., 2017).

#### 3.5.4 Cancer

Eleven positive effect sizes (*d* = 0.43 to *d* = 2.72) coming from six reviews were reported for nausea and vomiting, pain, and anxiety in cancer patients. Nine effects were significant (no information on the significance of the remaining two effects), one each can be interpreted as small or medium, and nine effects were large (including all effects on pain and anxiety; Figure 4).

#### 3.5.5 Irritable bowel syndrome

A group of 10 reviews examined the use of hypnosis for patients with irritable bowel syndrome. Effects were reported for gastrointestinal symptoms, but also for other issues that accompany the disease, e.g., pain, diarrhea, constipation, bloating/distension, mental distress, anxiety and depression, and health-related quality of life. For most of these outcomes, hypnosis revealed small (*i* = 11) or medium effects (*i* = 8). Only one effect size was large (abdominal pain; Lee et al., 2014). Range of effects was from *d* = 0.02 to *d* = 0.83, and eight of the 15 effects were significant (Figure 4).

#### 3.5.6 Smoking cessation

Five reviews (including three Cochrane reviews) compared hypnosis for smoking cessation to various control groups. Altogether, six effects were reported, ranging from *d* = 0.12 to *d* = 0.84 (Figure 5). Only two effects were significant. Two effects fell into the range of small effects, and one each was medium or large.

#### 3.5.7 Obesity

We included two reviews focusing on obesity reporting six effect sizes (range *d* = 0.09 to *d* = 1.58; Figure 5). In comparison to treatment as usual, attention control, or no treatment conditions, significant, large effects emerged for weight loss at post-treatment and follow-up. When hypnosis combined with cognitive-behavioral therapy (CBT) was compared to CBT alone, effects on weight loss were (very) small at post-treatment but increased to significant large effects at follow-up.

#### 3.5.8 Symptoms of mental/psychosomatic disorders

In seven reviews, nine effect sizes were reported for reduction of symptoms of mental or psychosomatic disorders. The effects ranged from *d* = 0.06 to *d* = 1.58, one effect was small, three were medium, and four were large. All effects were significant, except one non-significant null effect on anxiety symptoms when hypnosis in addition to CBT was compared to CBT alone (Figure 5).

#### 3.5.9 Various disorders

Moreover, one review (Flammer and Bongartz, 2003) reported a significant medium effect size of *d* = 0.64 for hypnosis in patients with somatic complaints on various outcomes. Three comprehensive reviews on hypnosis for various disorders pooled the effects of all included studies (Flammer and Bongartz, 2003; Eason and Parris, 2019; Ramondo et al., 2021). The four reported effect sizes were significant and ranged from *d* = 0.25 to *d* = 1.28. One can be regarded as small, two as medium, and another one as large.

#### 3.5.10 Harms or unintended effects of hypnosis

Eight of the 49 included reviews stated that no adverse events or side effects were reported in any of the included primary studies. One review (Schaefert et al., 2014) reported, that in one primary study one patient complained of slight dizziness after the first hypnosis session but continued the therapy, while in another study one dropout due to a panic attack during a hypnosis session was mentioned. In the review of Lam et al. (2015), only one study reported the incidence of adverse events, which was seen in the control group receiving zolpidem. The meta-analysis of Noergaard et al. (2019) considered “reduction in adverse events” as an outcome. Seven studies reported data, the pooled effect was in favor of hypnosis, but non-significant (*RR* = 0.61). In two reviews of hypnosis in labor and childbirth, there were two (Madden et al., 2012) or three primary studies (Madden et al., 2016) that reported data on any adverse events (i.e., maternal side effects, newborn resuscitation), but no significant differences between hypnosis and control groups were found. In two primary studies included in the review of Barnes et al. (2019), data on adverse events were reported without revealing any statistically significant difference between the hypnosis and control groups. The remaining 28 reviews did not include information on the safety of hypnosis (Supplementary Table S1).

#### 3.5.11 Moderators of efficacy

Reviews including *n* ≥ 10 studies were inspected for the reporting of moderator effects on the efficacy of hypnosis, potentially yielding sufficient statistical power to detect moderator effects in meta-regression or subgroup analyses. Results are summarized in Supplementary Table S4.

Various *patient characteristics* were examined in the reviews for their potential impact on the efficacy of hypnosis. Two reviews (Schnur et al., 2008; Chen et al., 2017) found significantly higher mean effect sizes in studies with children than in trials with adult populations, while Flammer and Alladin (2007) did not demonstrate differential effects based on age. Results on the impact of participants’ sex is mixed. One review reported significantly larger effects for studies with participants of mixed-sex compared to female-only populations (Chen et al., 2017). In contrast, another review could not find any differences between subgroups (male, female, mixed; Flammer and Alladin, 2007). In the review of Chen et al. (2017), significantly larger effects were found in studies with hematological malignancy than in trials with patients suffering from a solid tumor and for studies with procedure-related stressors compared to no such stressors. In reviews including patients undergoing surgery, neither effects of anesthesia (local, general; Tefikow et al., 2013; Holler et al., 2021), nor type of surgery (diagnostic procedure vs. other, Kekecs et al., 2014; Holler et al., 2021) could be found. Two reviews examined the impact of hypnotizability on treatment outcome, reporting medium (*r* = 0.31, Flammer and Alladin, 2007; *r* = 0.44; Flammer and Bongartz, 2003) and large correlations (*r* = 0.53; Milling et al., 2021) between hypnotizability and outcome.

Moderator analyses of *characteristics of the hypnosis intervention* included formal aspects of the intervention (format, setting, presentation mode, dose, frequency) and characteristics of the hypnosis intervention itself (type of hypnosis, standardization, use of direct suggestions). Neither format (hypnosis provided individually vs. in groups; Flammer and Alladin, 2007; Krouwel et al., 2021) nor setting (inpatients, outpatients, mixed; Flammer and Alladin, 2007) had an impact on the efficacy of hypnosis. Moderator analyses on the mode of presentation yielded significantly larger effects for live presentations in comparison to audio recorded presentations (Schnur et al., 2008; Kekecs et al., 2014). However, this was not the case in two other reviews (Tefikow et al., 2013; Holler et al., 2021). Results on the impact of dose on treatment outcome are also mixed. While two reviews found significant advantages for participants receiving more treatment time (Krouwel et al., 2021; Ramondo et al., 2021), two other reviews did not yield results to support differential effects of an intervention based on the dose (brief, medium, long; Tefikow et al., 2013; Holler et al., 2021). Moreover, there was no difference of frequency on outcome; weekly sessions produced effects similar to sessions less than once weekly; (Krouwel et al., 2021).

In the review of Flammer and Alladin (2007), type of hypnosis was demonstrated to be a moderator variable. “Modern hypnosis” was significantly more effective than classical hypnosis and mixed-forms of hypnosis. Furthermore, the impact of self-hypnosis, direct suggestions, and standardization was tested. Hypnosis was significantly more effective when it combined therapist delivery with self-hypnosis compared to self-hypnosis only (Chen et al., 2017) and hypnosis interventions had significantly larger effects when incorporating self-hypnosis than when self-hypnosis was not included (Milling et al., 2018). Studies that incorporated direct suggestions, i.e., suggestion directly addressing the primary outcome pain, did not produce larger effects than trials which did not use such suggestions (Milling et al., 2021). Additionally, Holler et al. (2021) found no effects of standardization of hypnosis.

*Characteristics of the control group* did not influence the size of reported effects, as similar results were reported for hypnosis compared to standard care and to attention control conditions (Schnur et al., 2008; Tefikow et al., 2013; Holler et al., 2021).

Finally, *characteristics of the included primary studies* had an influence on the reported effect size of hypnosis. In one review, sample size was significantly and inversely correlated with effect size (Schnur et al., 2008), another review found significantly larger effects for studies conducted in Europe than in trials conducted in America (Chen et al., 2017).

### 3.6 Reporting biases

In 37 of the included meta-analyses (75.5%), the authors carried out an adequate investigation of publication bias (small study bias) and discussed its likely impact on the results (Supplementary Table S3).

### 3.7 Certainty of evidence

Only few included reviews reported the certainty in the body of evidence. High certainty was reported only for two effects (self-reported pain, hypnosis vs. standard care, and vs. attention control; Jong et al., 2020). Certainty was moderate for five effects (pain intensity at post-treatment, hypnosis vs. control; Langlois et al., 2022; postoperative pain, postoperative anxiety, procedure time, and postoperative nausea and vomiting, hypnosis vs. control; Zeng et al., 2022). For four effects, the certainty of evidence was rated as low (spontaneous vaginal birth, hypnosis vs. control; Madden et al., 2016; smoking cessation, hypnosis vs. attention-matched behavioral treatments; Barnes et al., 2019; pain intensity follow-up and pain interference with daily activities, hypnosis vs. control; Langlois et al., 2022), and for five effects it was judged as very low (smoking cessation, hypnosis vs. no smoking cessation support; Hartmann-Boyce et al., 2021; self-reported pain, self-reported distress, and behavioral measures of distress, hypnosis vs. control; Birnie et al., 2018; use of pharmacological pain relief/anesthesia, hypnosis vs. control; Madden et al., 2016). Information on the inconsistency of results was reported more frequently in the reviews (*i* = 95, 80.5% of the extracted effect sizes). If reported, heterogeneity was low (*I*^2^ ≤ 50%) or significant for about one third of the effects (*i* = 32, 33.7%), in two thirds it was substantial (*I*^2^ > 50%) and/or significant (*i* = 63, 66.3%).

## 4 Discussion

With this systematic review of meta-analyses, we aimed at investigating the efficacy of clinical hypnosis interventions on various problem-relevant outcomes compared to non-active or active control groups. Through a comprehensive search in relevant databases, we identified 49 meta-analyses which were comprised of 261 distinct randomized controlled trials. Hypnosis interventions were examined for various mental and somatic health concerns and included adult populations as well as children and adolescents. The findings reported in the included meta-analyses underline the potential of hypnosis to positively impact various mental and somatic treatment outcomes. Specifically, more than half of the results were at least of medium effect size, and only one negative effect was reported (in fact, it was a null effect; *d* = −0.04). The most robust evidence was demonstrated in studies involving patients undergoing medical procedures with 12 reviews including 79 distinct primary studies, and in patients with pain (four reviews, 65 primary studies). The largest effects were seen for hypnosis in populations of children/adolescents, however, only four reviews focused on the efficacy of hypnosis specifically in children and/or adolescents. Of the 11 other meta-analyses that included children/adolescents and adult populations, one review supported the larger effects of hypnosis in children than in adults (Schnur et al., 2008), while two other meta-analyses did not find differences (Flammer and Alladin, 2007; Chen et al., 2017). The findings also demonstrated a substantial heterogeneity of primary study results in about two thirds of the reported effects, which clearly limits the generalizability of the findings and points to the need of exploring the variance in results via moderator analyses (Thompson, 1994). While most of the included meta-analyses did not involve a sufficient number of primary studies to allow for moderator analyses (Hedges and Pigott, 2004), nine meta-analyses investigated the impact of patient, intervention, and control group characteristics on the outcome of hypnosis (Flammer and Bongartz, 2003; Flammer and Alladin, 2007; Schnur et al., 2008; Tefikow et al., 2013; Kekecs et al., 2014; Chen et al., 2017; Milling et al., 2018, 2021; Ramondo et al., 2021). Overall, the moderator analyses tested a variety of potential impact factors but revealed mixed results. The only consistent evidence was reported for hypnotizability, which had medium to large positive effects on the outcome of hypnosis and supports findings of a meta-analysis on the impact of hypnotizability in clinical care settings (Montgomery et al., 2011). More specifically, 34 effects from 10 studies and 283 participants revealed statistically significant correlation (*r* = 0.24), indicating that greater hypnotizability was associated with greater effects of hypnosis interventions.

This synthesis led to the identification of some limitations of the evidence from the included systematic reviews and their primary studies. First, there is a large overlap of primary studies across the included reviews hampering an unbiased meta-synthesis of the reported effects. Second, heterogeneity of findings across the primary studies in the included reviews is mostly substantial, rendering it difficult to draw general conclusions and make clear recommendations for clinical practice. Third, a considerable number of effects is based on a very low number of studies/comparisons which negatively impacts the precision of the meta-analytic results (Brand and Bradley, 2016) and the possibility to examine moderator effects (Hedges and Pigott, 2004). Third, not only is the number of studies included in a considerable number of meta-analyses low, but also the number of patients per study. On the one hand, meta-analysis is advantageous in enhancing the precision of single studies by combining multiple findings to generate a pooled estimate (Finckh and Tramèr, 2008). On the other hand, if only a few (small) studies are included in an overall effect size, the precision of meta-analytic results may, nevertheless, be low (Guyatt et al., 2011). Fourth, most of the included reviews pooled their effects across various types of control groups (i.e., passive and active control groups), making it difficult to provide clear recommendations for clinical practice. Fifth, results of the included meta-analyses provided only sparse evidence on direct comparisons to other established interventions/interventions that have been proven efficacious (e.g., head-to-head comparisons). Finally, safety data were reported only in less than half of the reviews. To make a balanced decision about the use of hypnosis, it is essential to have comprehensive evidence on both benefits and harms. Therefore, systematic harm monitoring and reporting should become standard in RCTs of behavioral interventions (Klatte et al., 2023).

The results of our overview should be interpreted by considering some limitations of the review methods used. We did not calculate overall effects across the reported estimates, considering the substantial overlap of primary studies included in the meta-analyses. We further did not include the risk of bias within and across the primary studies as reported in the meta-analyses. Internal validity was assessed by using the Cochrane risk of bias tool (Higgins et al., 2011; Sterne et al., 2019) in most reviews (see Supplementary Table S1), but its impact on the effects was analyzed and reported not consistently in all reviews. Similarly, publication bias was not systematically examined in the included meta-analyses, potentially due to an insufficient number of primary studies to run tests for funnel plot asymmetry (with *k* < 10 the assessment methods are not very reliable; Dalton et al., 2016).

Our overview of meta-analyses carries various implications for clinical practice and for future research. Altogether, there is reasonable evidence from primary studies and systematic reviews that hypnosis can be an efficacious treatment option for patients with mental and somatic health problems. Although effects were heterogeneous for many outcomes, nearly all (99.2%) were positive, and the majority of effects was at least of medium effect size. Taking into account that clinical hypnosis is usually applied as a low-dose intervention, the results are promising. Hypnosis revealed the largest effects in children and for patients undergoing medical procedures. Because children and adolescents are often viewed as having higher average hypnotic ability than that of adults, it seems sensible that younger patients with mental or somatic health issues may be more responsive to hypnotic suggestions than adult patients (Accardi and Milling, 2009). Large effects of hypnosis in patients undergoing medical procedures might be driven by the patient-provider context and the idea that medical settings are especially conducive for responding to suggestions (Cheek, 1962; Varga, 2013).

Two of the included reviews focused on the effect of hypnosis in combination with CBT (Milling et al., 2018; Ramondo et al., 2021). The results suggest that hypnosis might have an additional impact when used as an adjunct to CBT, and the largest effects were seen when the hypnotic treatment directly integrated CBT principles into the hypnosis (Ramondo et al., 2021).

Our comprehensive overview also aimed at identifying research gaps to guide future research. In the light of our findings, we encourage hypnosis researchers to examine moderators of efficacy and to contribute further knowledge to the question: What works for whom and under which circumstances? We also emphasize the need for studies directly comparing hypnosis to established interventions that have been proven efficacious and to extend the knowledge basis on the effectiveness of hypnosis for children and adolescents. Finally, to allow for a balanced interpretation of benefits and harms of hypnosis and to derive clear recommendations for using hypnosis in various settings, harmful and unintended effects of hypnosis have to be explicitly and comprehensively assessed within future RCTs, transparently and completely reported in the respective publications, and should be considered in the reporting of subsequent meta-analyses.

## 5 Conclusion

This systematic review of meta-analyses provides a broad overview of the substantial evidence supporting the use of clinical hypnosis to treat a range of mental and somatic health issues. The vast majority (99.2%) of outcomes demonstrated positive effects, with over half exhibiting at least a medium effect size. Notably, the largest effects were found when hypnosis was used with child/adolescent patient populations, to treat pain, and to aid medical procedures. The review also revealed important limitations, including substantial heterogeneity in study outcomes which warrants a call for more studies directly comparing hypnosis to established interventions. Greater awareness for assessing adverse events in efficacy research on hypnosis is needed. Overall, these findings support the use of hypnosis in clinical practice and mental health professionals and medical providers are thus encouraged to consider the use and referral of hypnosis interventions, especially for patients undergoing medical procedures, those experiencing pain, and when working with children.

## Data availability statement

The data analyzed in this study is subject to the following licenses/restrictions: the data that support the findings of this study are available from the corresponding author upon reasonable request. Requests to access these datasets should be directed to jenny.rosendahl@med.uni-jena.de.

## Author contributions

JR: Conceptualization, Data curation, Formal analysis, Investigation, Methodology, Project administration, Visualization, Writing – original draft. CA: Data curation, Investigation, Visualization, Writing – original draft, Writing – review & editing. AH: Data curation, Formal analysis, Investigation, Writing – original draft.
