# Supplementary material for: Meta-analytic evidence on the efficacy of hypnosis for mental and somatic health issues: a 20-year perspective
Source: Front Psychol. 2024 Jan 8;14:1330238. doi: 10.3389/fpsyg.2023.1330238 (PMC10807512; doi:10.3389/fpsyg.2023.1330238)
Supplement: Supplementary file 3 [file Data_Sheet_1.PDF]

Supplementary Material

Supplementary material 1. Full search strategy

MEDLINE Search Strategy (last search on 06.03.2023)

#	Search term(s)
1	“hypnosis”
2	“hypnotherapeutic”
3	“hypnotherapy”
4	“hypnotised”
5	#1 or #2 or #3 or #4
6	“randomized controlled trial” or “controlled clinical trial“ or “randomized“
7	“randomly” [Title/Abstract]
8	“trial” [Title/Abstract]
9	#6 or #7 or #8
10	“meta-analysis“ or “metaanalysis” or “systematic review“
11	#5 and #9 and #10
12	limit 11 to yr="2000 – 2023“

PubMed Search Strategy (last search on 09.03.2023)

#	Search term(s)
1	hypnosis
2	Hypnotherapeutic
3	hypnotherapy
4	hypnotised
5	#1 OR #2 OR #3 OR #4
6	(randomized controlled trial) OR (controlled clinical trial) OR (randomized)
7	randomly[Title/Abstract]
8	trial [Title/Abstract]

9	#6 OR #7 OR #8
10	(meta-analysis) OR (systematic review)
11	#5 AND #9 AND #10
12	("2000/01/01"[Date - Publication] : "3000"[Date - Publication]) AND #11

Cochrane Library search strategy (last search on 09.03.2023)

#	Search term(s)
1	(hypnosis):ti,ab,kw OR (hypnotherapy):ti,ab,kw OR (hypnotherapeutic):ti,ab,kw OR (hypnotised):ti,ab,kw
2	Hypnosis [MeSH]
3	#1 OR #2
4	(randomized controlled trial):ti,ab,kw OR (controlled clinical trial):ti,ab,kw OR (randomized):ti,ab,kw OR (randomly):ti,ab,kw OR (trial):ti,ab,kw
5	(systematic review):ti,ab,kw OR (meta-analysis):ti,ab,kw OR (meta analysis):ti,ab,kw OR (metaanalysis):ti,ab,kw
6	Meta analysis as topic[MeSH]
7	Systematic review as topic[MeSH]
8	#5 OR #6 OR #7
9	#3 AND #4 AND #8
10	Custom range: 01/01/2000-01/03/2023 AND #9

APA PsycInfo search strategy (last search on 02.03.2023)

#	Search term(s)
1	hypnosis
2	hypnotherapeutic
3	hypnotherapy
4	Hypnotised
5	S1 OR S2 OR S3 OR S4
6	(randomized controlled trial) OR (controlled clinical trial OR (randomized)
7	TI randomly OR AB randomly
8	TI trial OR AB trial

9	S6 OR S7 OR S8
10	(meta-analysis or systematic review) OR meta-analysis OR metaanalysis OR meta analysis OR systematic review OR systematic review [MeSH] OR meta-analysis [MeSH] OR meta-analysis or systematic review [MeSH]
11	S5 AND S9 AND S10
12	PY 2000 - 2023 AND S11

Web of Science search strategy (last search on 09.03.2023)

#	Search term(s)
1	ALL=(hypnosis)
2	ALL=(hypnotherapeutic)
3	ALL=(hypnotherapy)
4	ALL=(hypnotised)
5	#1 OR #2 OR #3 OR #4
6	(ALL=(randomized controlled trial)) OR ALL=(randomised controlled trial)
7	ALL=(controlled clinical trial)
8	(ALL=(randomized)) OR ALL=(randomised)
9	(TI=(randomly)) OR AB=(randomly)
10	(TI=(trial)) OR AB=(trial)
11	#6 OR #7 OR #8 OR #9 OR #10
12	AK=(meta analysis) OR TS=(meta analysis) OR ALL=(meta analysis) OR ALL=(meta-analysis) OR ALL=(metaanalysis) OR (TI=(meta analysis)) OR AB=(meta analysis) OR (TI=(meta-analysis)) OR AB=(meta-analysis) OR (TI=(metaanalysis)) OR AB=(metaanalysis)
13	DT=(Review) OR ALL=(systematic review) OR #12
14	#5 AND #11 AND #13
15	Timespan: 2000-01-01 to 2023-03-01 AND #14

Health Technology Assessment (HTA) Database search strategy (last search on 06.03.2023)

#	Search term(s)
1	(hypnosis) OR (hypnotherapeutic) OR (hypnotherapy) OR (hypnotised) OR (hypnosis)[mh]
2	(randomized controlled trial) OR (controlled clinical trial) OR (randomized)

3	Randomly[title] or randomly[abstract]
4	Trial[title] or trial[abstract]
5	((Randomly[title] or randomly[abstract])) OR ((randomized controlled trial) OR (controlled clinical trial) OR (randomized)) OR (Trial[title] or trial[abstract])
6	(meta-analysis) OR (metaanalysis) OR (systematic review) OR (meta-analysis)[mh] OR (systematic review)[mh]
7	1 AND 5 AND 6

Database of Abstracts of Reviews of Effects (DARE) search strategy (last search on 06.03.2023)

#	Search term(s)
1	Hypnosis
2	Hypnotherapy
3	Hypnotised
4	#1 OR #2 OR #3
5	(randomized controlled trial) OR (controlled clinical trial) OR (randomized)
6	Randomly[title] OR Trial[title]
7	#5 OR #6
8	(meta-analysis) OR (metaanalysis) OR (systematic review)
9	5 AND 9 AND 10
10	(#9) FROM 2000 TO 2023

Scopus search strategy (last search on 09.03.2023)

#	Search term(s)
1	Hypnosis
2	Hypnotherapeutic
3	Hypnotherapy
4	Hypnotised
5	#1 OR #2 OR #3 OR #4<br>ALL (hypnosis) OR (ALL (hypnotherapeutic)) OR (ALL (hypnotherapy)) OR (ALL (hypnot ised))
6	ALL (randomized AND controlled AND trial) OR ALL (controlled AND clinical AND trial) OR ALL (randomized)

7	TITLE-ABS-KEY (randomly)
8	TITLE-ABS-KEY (trial)
9	#6 OR #7 OR #8<br>ALL (randomized AND controlled AND trial) OR (ALL (controlled AND clinical AND trial)) OR (ALL (randomized)) OR (TITLE-ABS-KEY (randomly)) OR (TITLE-ABS-KEY (trial))
10	TITLE-ABS-KEY (systematic AND review) OR TITLE-ABS-KEY (meta-analysis) OR TITLE-ABS-KEY (metaanalysis) OR TITLE-ABS-KEY (meta AND analysis)
11	#5 AND #9 AND #10
12	PUBYEAR>1999

Supplementary material 2. References of the included systematic reviews

Barnes, J., Dong, C. Y., McRobbie, H., Walker, N., Mehta, M., & Stead, L. F. (2010). Hypnotherapy for smoking cessation. *Cochrane Database of Systematic Reviews, 10*, CD001008. https://doi.org/10.1002/14651858.CD001008.pub2

Barnes, J., McRobbie, H., Dong, C. Y., Walker, N., & Hartmann-Boyce, J. (2019). Hypnotherapy for smoking cessation. *Cochrane Database of Systematic Reviews, 6*, CD001008. https://doi.org/10.1002/14651858.CD001008.pub3

Birnie, K. A., Noel, M., Chambers, C. T., Uman, L. S., & Parker, J. A. (2018). Psychological interventions for needle-related procedural pain and distress in children and adolescents. *Cochrane Database of Systematic Reviews*, *10*, CD005179. https://doi.org/10.1002/14651858.CD005179.pub4

Black, C. J., Thakur, E. R., Houghton, L. A., Quigley, E. M. M., Moayyedi, P., & Ford, A. C. (2020). Efficacy of psychological therapies for irritable bowel syndrome: Systematic review and network meta-analysis. *Gut, 69*(8), 1441-1451. https://doi.org/10.1136/gutjnl-2020-321191

Burghardt, S., Koranyi, S., Magnucki, G., Strauss, B., & Rosendahl, J. (2018). Non-pharmacological interventions for reducing mental distress in patients undergoing dental procedures: Systematic review and meta-analysis. *Journal of Dentistry*, *69*, 22-31. https://doi.org/10.1016/j.jdent.2017.11.005

Chen, P. Y., Liu, Y. M., & Chen, M. L. (2017). The effect of hypnosis on anxiety in patients with cancer: A meta-analysis. *Worldviews on Evidence-Based Nursing*, *14*(3), 223-236. https://doi.org/10.1111/wvn.12215

Cyna, A. M., McAuliffe, G. L., & Andrew, M. I. (2004). Hypnosis for pain relief in labour and childbirth: A systematic review. *British Journal of Anaesthesia*, *93*(4), 505–511. https://doi.org/10.1093/bja/aeh225

Danon, N., Al-Gobari, M., Burnand, B., & Rodondi, P. Y. (2022). Are mind-body therapies effective for relieving cancer-related pain in adults? A systematic review and meta-analysis. *Psycho-Oncology*, *31*(3), 345–371. https://doi.org/10.1002/pon.5821

Eason, A. D., & Parris, B. A. (2019). Clinical applications of self-hypnosis: A systematic review and meta-analysis of randomized controlled trials. *Psychology of Consciousness: Theory, Research, and Practice*, *6*(3), 262-278. https://doi.org/10.1037/cns0000173

Flammer, E., & Alladin, A. (2007). The efficacy of hypnotherapy in the treatment of psychosomatic disorders: Meta-analytical evidence. *International Journal of Clinical and Experimental Hypnosis*, *55*(3), 251–274. https://doi.org/10.1080/00207140701338696

Flammer, E., & Bongartz, W. (2003). On the efficacy of hypnosis: A meta-analytic study. *Contemporary Hypnosis*, *20*(4), 179-197. https://doi.org/10.1002/ch.277

Ford, A. C., Lacy, B. E., Harris, L. A., Quigley, E. M. M., & Moayyedi, P. (2019). Effect of Antidepressants and psychological therapies in irritable bowel syndrome: An updated systematic review and meta-analysis. *American Journal of Gastroenterology, 114*(1), 21-39. https://doi.org/10.1038/s41395-018-0222-5

Garland, E. L., Brintz, C. E., Hanley, A. W., Roseen, E. J., Atchley, R. M., Gaylord, S. A., Faurot, K. R., Yaffe, J., Fiander, M., & Keefe, F. J. (2020). Mind-body therapies for opioid-treated pain: A systematic review and meta-analysis. *JAMA Internal Medicine, 180*(1), 91-105. https://doi.org/10.1001/jamainternmed.2019.4917

Hartmann-Boyce, J., Livingstone-Banks, J., Ordóñez-Mena, J. M., Fanshawe, T. R., Lindson, N., Freeman, S. C., Sutton, A. J., Theodoulou, A., & Aveyard, P. (2021). Behavioural interventions for smoking cessation: An overview and network meta-analysis. *Cochrane Database of Systematic Reviews, 1*, CD013229. https://doi.org/10.1002/14651858.CD013229.pub2

Henrich, J. F., Knittle, K., De Gucht, V., Warren, S., Dombrowski, S. U., & Maes, S. (2015). Identifying effective techniques within psychological treatments for irritable bowel syndrome: A meta-analysis. *Journal of Psychosomatic Research, 78*(3), 205-222. https://doi.org/10.1016/j.jpsychores.2014.12.009

Holler, M., Koranyi, S., Strauss, B., & Rosendahl, J. (2021). Efficacy of hypnosis in adults undergoing surgical procedures: A meta-analytic update. *Clinical Psychology Review, 85*, 102001. https://doi.org/10.1016/j.cpr.2021.102001

Jong, M. C., Boers, I., van Wietmarschen, H., Busch, M., Naafs, M. C., Kaspers, G. J. L., & Tissing, W. J. E. (2020). Development of an evidence-based decision aid on complementary and alternative medicine (CAM) and pain for parents of children with cancer. *Supportive Care in Cancer*, *28*(5), 2415-2429. https://doi.org/10.1007/s00520-019-05058-8

Kekecs, Z., Nagy, T., & Varga, K. (2014). The effectiveness of suggestive techniques in reducing postoperative side effects: A meta-analysis of randomized controlled trials. *Anesthesia & Analgesia*, *119*(6), 1407-1419. https://doi.org/ANE.0000000000000466

Krouwel, M., Farley, A., Greenfield, S., Ismail, T., & Jolly, K. (2021). Systematic review, meta-analysis with subgroup analysis of hypnotherapy for irritable bowel syndrome, effect of intervention characteristics. *Complementary Therapies in Medicine, 57*, 102672. https://doi.org/10.1016/j.ctim.2021.102672

Laird, K. T., Tanner-Smith, E. E., Russell, A. C., Hollon, S. D., & Walker, L. S. (2016). Short-term and long-term efficacy of psychological therapies for irritable bowel syndrome: A systematic review and meta-analysis. *Clinical Gastroenterology and Hepatology, 14*(7), 937-947.e4. https://doi.org/10.1016/j.cgh.2015.11.020

Laird, K. T., Tanner-Smith, E. E., Russell, A. C., Hollon, S. D., & Walker, L. S. (2017). Comparative efficacy of psychological therapies for improving mental health and daily functioning in irritable bowel syndrome: A systematic review and meta-analysis. *Clinical Psychology Review*, *51*, 142-152. https://doi.org/10.1016/j.cpr.2016.11.001

Lam, T. H., Chung, K. F., Yeung, W. F., Yu, B. Y., Yung, K. P., & Ng, T. H. (2015). Hypnotherapy for insomnia: A systematic review and meta-analysis of randomized controlled trials. *Complementary Therapies in Medicine, 23*(5), 719-732. https://doi.org/10.1016/j.ctim.2015.07.011

Langlois, P., Perrochon, A., David, R., Rainville, P., Wood, C., Vanhaudenhuyse, A., Pageaux, B., Ounajim, A., Lavalliere, M., Debarnot, U., Luque-Moreno, C., Roulaud, M., Simoneau, M., Goudman, L., Moens, M., Rigoard, P., & Billot, M. (2022). Hypnosis to manage musculoskeletal

and neuropathic chronic pain: A systematic review and meta-analysis. *Neuroscience & Biobehavioral Reviews*, *135*, 104591. https://doi.org/10.1016/j.neubiorev.2022.104591

Lee, H. H., Choi, Y. Y., & Choi, M. G. (2014). The efficacy of hypnotherapy in the treatment of irritable bowel syndrome: A systematic review and meta-analysis. *Journal of Neurogastroenterology and Motility*, *20*(2), 152-162. https://doi.org/10.5056/jnm.2014.20.2.152

Madden, K., Middleton, P., Cyna, A. M., Matthewson, M., & Jones, L. (2012). Hypnosis for pain management during labour and childbirth. *Cochrane Database of Systematic Reviews, 11*, CD009356. https://doi.org/10.1002/14651858.CD009356.pub2

Madden, K., Middleton, P., Cyna, A. M., Matthewson, M., & Jones, L. (2016). Hypnosis for pain management during labour and childbirth. *Cochrane Database of Systematic Reviews*, *5*, CD009356. https://doi.org/10.1002/14651858.CD009356.pub3

Milling, L. S., Gover, M. C., & Moriarty, C. L. (2018). The effectiveness of hypnosis as an intervention for obesity: A meta-analytic review. *Psychology of Consciousness: Theory Research, and Practice*, *5*(1), 29-45. https://doi.org/10.1037/cns0000139

Milling, L. S., Valentine, K. E., LoStimolo, L. M., Nett, A. M., & McCarley, H. S. (2021). Hypnosis and the alleviation of clinical pain: A comprehensive meta-analysis. *International Journal of Clinical and Experimental Hypnosis*, *69*(3), 297-322. https://doi.org/10.1080/00207144.2021.1920330

Noergaard, M. W., Håkonsen, S. J., Bjerrum, M., & Pedersen, P. U. (2019). The effectiveness of hypnotic analgesia in the management of procedural pain in minimally invasive procedures: A systematic review and meta-analysis. *Journal of Clinical Nursing*, *28*(23-24), 4207-4224. https://doi.org/10.1111/jocn.15025

Nunns, M., Mayhew, D., Ford, T., Rogers, M., Curle, C., Logan, S., & Moore, D. (2018). Effectiveness of nonpharmacological interventions to reduce procedural anxiety in children and adolescents undergoing treatment for cancer: A systematic review and meta-analysis. *Psycho-Oncology*, *27*(8), 1889-1899. https://doi.org/10.1002/pon.4749

O'Toole, S. K., Solomon, S. L., & Bergdahl, S. A. (2016). A meta-analysis of hypnotherapeutic techniques in the treatment of PTSD symptoms. *Journal of Traumatic Stress*, *29*(1), 97-100. https://doi.org/10.1002/jts.22077

Peng, W. Y., Ye, K., Qin, D., Tang, T. C., Chen, M., & Zheng, H. (2021). Searching for a definition of refractory irritable bowel syndrome: A systematic review and meta-analysis. *Journal of Gastrointestinal & Liver Diseases*, *30*(4), 495-505. https://doi.org/10.15403/jgld-3952

Provençal, S. C., Bond, S., Rizkallah, E., & El-Baalbaki, G. (2018). Hypnosis for burn wound care pain and anxiety: A systematic review and meta-analysis. *Burns*, *44*(8), 1870-1881. https://doi.org/10.1016/j.burns.2018.04.017

Ramondo, N., Gignac, G. E., Pestell, C. F., & Byrne, S. M. (2021). Clinical hypnosis as an adjunct to cognitive behavior therapy: An updated meta-analysis. *International Journal of Clinical and Experimental Hypnosis*, *69*(2), 169-202. https://doi.org/10.1080/00207144.2021.1877549

Richardson, J., Smith, J. E., McCall, G., Richardson, A., Pilkington, K., & Kirsch, I. (2007). Hypnosis for nausea and vomiting in cancer chemotherapy: A systematic review of the research

evidence. *European Journal of Cancer Care, 16*(5), 402–412. https://doi.org/10.1111/j.1365-2354.2006.00736.x

Rotaru, T. S., & Rusu, A. (2016). A meta-analysis for the efficacy of hypnotherapy in alleviating PTSD symptoms. *International Journal of Clinical and Experimental Hypnosis, 64*(1), 116-136. https://doi.org/10.1080/00207144.2015.1099406

Schaefert, R., Klose, P., Moser, G., & Hauser, W. (2014). Efficacy, tolerability, and safety of hypnosis in adult irritable bowel syndrome: Systematic review and meta-analysis. *Psychosomatic Medicine, 76*(5), 389-398. https://doi.org/10.1097/PSY.0000000000000039

Scheffler, M., Koranyi, S., Meissner, W., Strauss, B., & Rosendahl, J. (2018). Efficacy of non-pharmacological interventions for procedural pain relief in adults undergoing burn wound care: A systematic review and meta-analysis of randomized controlled trials. *Burns, 44*(7), 1709-1720. https://doi.org/10.1016/j.burns.2017.11.019

Schnur, J. B., Kafer, I., Marcus, C., & Montgomery, G. H. (2008). Hypnosis to manage distress related to medical procedures: A meta-analysis. *Contemporary Hypnosis, 25*(3-4), 114-128. https://doi.org/10.1002%2Fch.364

Shah, K., Ramos-Garcia, M., Bhavsar, J., & Lehrer, P. (2020). Mind-body treatments of irritable bowel syndrome symptoms: An updated meta-analysis. *Behaviour Research & Therapy, 128*, 103462. https://doi.org/10.1016/j.brat.2019.103462

Shih, M., Yang, Y. H., & Koo, M. (2009). A meta-analysis of hypnosis in the treatment of depressive symptoms: A brief communication. *International Journal of Clinical and Experimental Hypnosis, 57*(4), 431-442. https://doi.org/10.1080/00207140903099039

Smith, C. A., Collins, C. T., Cyna, A. M., & Crowther, C. A. (2003). Complementary and alternative therapies for pain management in labour. *Cochrane Database of Systematic Reviews, 2*, CD003521. https://doi.org/10.1002/14651858.CD003521

Smith, C. A., Collins, C. T., Cyna, A. M., & Crowther, C. A. (2006). Complementary and alternative therapies for pain management in labour. *Cochrane Database of Systematic Reviews, 4*, CD003521. https://doi.org/10.1002/14651858.CD003521.pub2

Tahiri, M., Mottillo, S., Joseph, L., Pilote, L., & Eisenberg, M. J. (2012). Alternative smoking cessation aids: A meta-analysis of randomized controlled trials. *American Journal of Medicine, 125*(6), 576-584. https://doi.org/10.1016/j.amjmed.2011.09.028

Tefikow, S., Barth, J., Maichrowitz, S., Beelmann, A., Strauss, B., & Rosendahl, J. (2013). Efficacy of hypnosis in adults undergoing surgery or medical procedures: A meta-analysis of randomized controlled trials. *Clinical Psychology Review, 33*(5), 623-636. https://doi.org/10.1016/j.cpr.2013.03.005

Uman, L. S., Chambers, C. T., McGrath, P. J., & Kisely, S. (2006). Psychological interventions for needle-related procedural pain and distress in children and adolescents. *Cochrane Database of Systematic Reviews, 4*, CD005179. https://doi.org/10.1002/14651858.CD005179.pub2

Uman, L. S., Birnie, K. A., Noel, M., Parker, J. A., Chambers, C. T., McGrath, P. J., & Kisely, S. R. (2013). Psychological interventions for needle-related procedural pain and distress in children and adolescents. *Cochrane database of systematic reviews, 10*, CD005179. https://doi.org/10.1002/14651858.CD005179.pub3

Zech, N., Hansen, E., Bernardy, K., & Hauser, W. (2017). Efficacy, acceptability and safety of guided imagery/hypnosis in fibromyalgia: A systematic review and meta-analysis of randomized controlled trials. *European Journal of Pain*, *21*(2), 217-227. https://doi.org/10.1002/ejp.933

Zeng, J., Wang, L., Cai, Q., Wu, J., & Zhou, C. (2022). Effect of hypnosis before general anesthesia on postoperative outcomes in patients undergoing minor surgery for breast cancer: A systematic review and meta-analysis. *Gland Surgery*, *11*(3), 588-598. https://doi.org/10.21037/gs-22-114

Supplementary Material 3. Excluded studies with the main reason for exclusion and references

**Study**	**Reason for exclusion**
Birnie et al. (2014)	Duplicate publication of Uman et al. (2013)
Bongartz et al. (2002)	Duplicate publication of Flammer & Bongartz 2003
Gordon et al. (2022)	$k \leq 3$ studies included in effect size estimates
Hu et al. (2021)	No results for hypnosis-subgroup reported
Miller et al. (2001)	Included also studies in healthy participants
Montgomery et al. (2011)	Hypnotic suggestibility as outcome, not effects of hypnosis
Powell et al. (2016)	$k \leq 3$ studies included in effect size estimates
Tao et al. (2017)	$k \leq 3$ studies included in effect size estimates
Thompson et al. (2019)	Meta-analysis of controlled experimental trials, no RCTs
Uman et al. (2008)	Duplicate publication of Uman et al. (2006)
Webb et al. (2007)	$k \leq 3$ studies included in effect size estimates

References

Birnie, K. A., Noel, M., Parker, J. A., Chambers, C. T., Uman, L. S., Kisely, S. R., & McGrath, P. J. (2014). Systematic review and meta-analysis of distraction and hypnosis for needle-related pain and distress in children and adolescents. *Journal of Pediatric Psychology, 39*(8), 783-808. https://doi.org/10.1093/jpepsy/jsu029

Bongartz, W., Flammer, E., & Schwonke, R. (2002). Die Effektivität der Hypnose: Eine meta-analytische Studie [Efficiency of hypnosis: A meta-analytic study]. *Psychotherapeut, 47*(2), 67-76. https://doi.org/10.1007/s00278-002-0207-z

Gordon, M., Sinopoulou, V., Tabbers, M., Rexwinkel, R., de Bruijn, C., Dovey, T., Gasparetto, M., Vanker, H., & Benninga, M. (2022). Psychosocial Interventions for the Treatment of Functional Abdominal Pain Disorders in Children: A Systematic Review and Meta-analysis. *JAMA Pediatrics*, *176*(6), 560–568. https://doi.org/10.1001/jamapediatrics.2022.0313

Hu, Y., Lu, H., Huang, J., & Zang, Y. (2021). Efficacy and safety of non-pharmacological interventions for labour pain management: A systematic review and Bayesian network meta-analysis. *Journal of Clinical Nursing, 30*(23-24), 3398–3414. https://doi.org/10.1111/jocn.15865

Miller, G. E., & Cohen, S. (2001). Psychological interventions and the immune system: a meta-analytic review and critique. *Health Psychology*, *20*(1), 47–63. https://doi.org/10.1037//0278-6133.20.1.47

Montgomery, G. H., Schnur, J. B., & David, D. (2011). The impact of hypnotic suggestibility in clinical care settings. *International Journal of Clinical and Experimental Hypnosis*, *59*(3), 294–309. https://doi.org/10.1080/00207144.2011.570656

Powell, R., Scott, N. W., Manyande, A., Bruce, J., Vogele, C., Byrne-Davis, L. M., Unsworth, M., Osmer, C., & Johnston, M. (2016). Psychological preparation and postoperative outcomes for adults undergoing surgery under general anaesthesia. *Cochrane Database of Systematic Reviews, 5*, CD008646. https://doi.org/https://dx.doi.org/10.1002/14651858.CD008646.pub2

Tao, W. W., Tao, X. M., & Song, C. L. (2017). Effects of non-pharmacological supportive care for hot flushes in breast cancer: a meta-analysis. *Supportive Care in Cancer, 25*(7), 2335–2347. https://doi.org/10.1007/s00520-017-3691-y

Thompson, T., Terhune, D. B., Oram, C., Sharangparni, J., Rouf, R., Solmi, M., Veronese, N., & Stubbs, B. (2019). The effectiveness of hypnosis for pain relief: A systematic review and meta-analysis of 85 controlled experimental trials. *Neuroscience and Biobehavioral Reviews, 99*, 298–310. https://doi.org/10.1016/j.neubiorev.2019.02.013

Uman, L. S., Chambers, C. T., McGrath, P. J., & Kisely, S. (2008). A systematic review of randomized controlled trials examining psychological interventions for needle-related procedural pain and distress in children and adolescents: An abbreviated Cochrane review. *Journal of Pediatric Psychology, 33*(8), 842–854. https://doi.org/10.1093/jpepsy/jsn031

Webb, A. N., Kukuruzovic, R. H., Catto-Smith, A. G., & Sawyer, S. M. (2007). Hypnotherapy for treatment of irritable bowel syndrome. *Cochrane Database of Systematic Reviews, 4*, CD005110. https://doi.org/10.1002/14651858.CD005110.pub2
