# Supplementary material for: Meta-analytic evidence on the efficacy of hypnosis for mental and somatic health issues: a 20-year perspective
Source: Front Psychol. 2024 Jan 8;14:1330238. doi: 10.3389/fpsyg.2023.1330238 (PMC10807512; doi:10.3389/fpsyg.2023.1330238)

Supplementary Tables and Figures

Supplementary Table 1. Detailed characteristics of the included reviews

--- Provided as separate file ---

Supplementary Table 2. Primary studies included in the systematic reviews and overlap

--- Provided as separate file ---

Supplementary Table 3. Quality assessment of each included review. Critical items of the AMSTAR 2 tool and overall rating.

	Review	Overall rating	Review protocol	Comprehensive literature search strategy	List of excluded studies and justification of exclusion	Satisfactory risk of bias assessment of included studies	Appropriate methods for meta-analysis	Account for risk of bias in interpretation/ discussion	Publication bias assessed and discussed
Medical procedures	Birnie et al. 2014	High	Yes	Yes	Yes	Yes	Yes	Yes	Yes
	Birnie et al. 2018	High	Yes	Yes	Yes	Yes	Yes	Yes	Yes
	Burghardt et al. 2018	Low	Yes	Yes	No	Yes	Yes	Yes	Yes
	Holler et al. 2021	Low	Yes	Yes	No	Yes	Yes	Yes	Yes
	Kekecs et al. 2014	Critically Low	No	Yes	No	Yes	Yes	Yes	Yes
	Noergaard et al. 2019	Critically Low	Yes	Yes	No	Yes	Yes	No	Yes
	Provençal et al. 2018[a]	Moderate	Yes	Yes	Yes	Yes	Yes	Yes	Yes
	Scheffler et al. 2018	Low	Yes	Yes	No	Yes	Yes	Yes	Yes
	Schnur et al. 2008	Critically Low	No	Yes	No	No	Yes	No	Yes
	Tefikow et al. 2013	Low	Yes	Yes	No	Yes	Yes	Yes	Yes
	Uman et al. 2006	High	Yes	Yes	Yes	Yes	Yes	Yes	Yes
	Zeng et al. 2022	Critically Low	No	Yes	No	Yes	Yes	Yes	Yes
Labor and childbirth	Cyna et al. 2004	Critically Low	No	Yes	No	No	No	No	No
	Madden et al. 2012	High	Yes	Yes	Yes	Yes	Yes	Yes	Yes
	Madden et al. 2016	High	Yes	Yes	Yes	Yes	Yes	Yes	Yes
	Smith et al. 2003	Low	Yes	Yes	Yes	Yes	Yes	Yes	No
	Smith et al. 2006	Low	Yes	Yes	Yes	Yes	Yes	Yes	No
Pain	Garland et al. 2020	Critically Low	No	Yes	No	Yes	No	Yes	Yes
	Langlois et al. 2022[b]	High	Yes	Yes	Yes	Yes	Yes	Yes	Yes
	Milling et al. 2021	Critically Low	No	Yes	No	Yes	Yes	Yes	Yes
	Zech et al. 2017	Low	Yes	Yes	No	Yes	Yes	Yes	Yes
Cancer	Chen et al. 2017	Critically Low	No	Yes	No	No	Yes	No	Yes
	Danon et al. 2021	Low	Yes	Yes	No	Yes	Yes	Yes	Yes
	Jong et al. 2020	Critically Low	No	Yes	No	Yes	No	No	No
	Nunns et al. 2018	Low	Yes	Yes	Yes	Yes	Yes	Yes	No
	Richardson et al. 2007	Critically Low	No	Yes	Yes	No	No	No	No

	Review	Overall rating	Review protocol	Comprehensive literature search strategy	List of excluded studies and justification of exclusion	Satisfactory risk of bias assessment of included studies	Appropriate methods for meta-analysis	Account for risk of bias in interpretation/ discussion	Publication bias assessed and discussed
Irritable bowel syndrome	Black et al. 2020	Low	Yes	Yes	No	Yes	Yes	Yes	Yes
	Ford et al. 2019	Critically Low	No	Yes	No	Yes	Yes	Yes	Yes
	Henrich et al. 2015	Critically Low	No	Yes	No	Yes	Yes	No	No
	Krouwel et al. 2021	Critically Low	Yes	Yes	No	Yes	Yes	No	No
	Laird et al. 2016	Low	Yes	Yes	No	Yes	Yes	Yes	Yes
	Laird et al. 2017	Critically Low	No	Yes	No	Yes	Yes	Yes	Yes
	Lee et al. 2014	Critically Low	No	Yes	No	Yes	No	No	Yes
	Peng et al. 2021	Critically Low	No	Yes	No	Yes	No	Yes	No
	Schaefert et al. 2014	Low	No	Yes	Yes	Yes	Yes	Yes	Yes
	Shah et al. 2020	Critically Low	No	No	No	No	No	No	No
Smoking cessation	Barnes et al. 2010	High	Yes	Yes	Yes	Yes	Yes	Yes	Yes
	Barnes et al. 2019	High	Yes	Yes	Yes	Yes	Yes	Yes	Yes
	Hartmann-Boyce et al. 2021	High	Yes	Yes	Yes	Yes	Yes	Yes	Yes
	Tahiri et al. 2012	Critically Low	No	Yes	No	Yes	No	No	No
Obes	Milling et al. 2018	Critically Low	No	Yes	No	Yes	Yes	Yes	Yes
Symptoms of mental/ psychosom. disorders	Flammer et al. 2007	Critically Low	No	Yes	No	No	Yes	No	Yes
	Lam et al. 2015	Critically Low	No	Yes	No	Yes	No	No	No
	O'Toole et al. 2016	Critically Low	No	Yes	No	Yes	No	No	Yes
	Rotaru et al. 2016	Critically Low	No	Yes	No	No	No	No	Yes
	Shih et al. 2009	Critically Low	No	Yes	No	No	No	No	Yes
Various	Flammer & Bongartz 2003	Critically Low	No	Yes	No	No	No	No	Yes
	Eason & Parris 2019	Critically Low	No	Yes	No	No	Yes	No	Yes
	Ramondo et al. 2021	Low	Yes	Yes	No	Yes	Yes	Yes	Yes

Obes, Obesity; Psychosom., Psychosomatic; Various, Various disorders; [a] Two non-critical weaknesses (review did not explain the selection of the study designs for inclusion and did not assess potential impact of risk of bias on results); [b] One non-critical weakness (review did not assess potential impact of risk of bias on results)

Supplementary Table 4. Moderator effects on the efficacy of hypnosis reported by the included reviews

Moderator variables	Moderator effects	Review
**Patient characteristics**		
Children	Children > adults	Schnur et al. 2008
	Children/adolescents = adults = mixed	Flammer & Alladin 2007
	Children = adults	Chen et al. 2017
Sex	Mixed sex > female only	Chen et al. 2017
	Male = female = mixed	Flammer & Alladin 2007
Procedure	Procedure-related stressors > no procedure-related stressors	Chen et al. 2017
	Local anesthesia = general anesthesia	Tefikow et al. 2013; Holler et al. 2021
	Diagnostic procedure = other	Holler et al. 2021
	Major surgery = minor surgery	Kekecs et al. 2014
**Characteristics of the hypnosis intervention**		
Format	Individual = group	Flammer & Alladin 2007; Krouwel et al. 2021
Mode of presentation	Face-to-face setting = pre-recorded tape	Tefikow et al. 2013; Holler et al. 2021
	Live presentation > recorded presentation	Kekecs et al. 2014; Schnur et al. 2008
Setting	Inpatient = outpatient = mixed	Flammer & Alladin 2007
Dose	Higher dose > lower dose	Krouwel et al. 2021; Ramondo et al. 2021
	Higher dose = lower dose	Tefikow et al. 2013; Holler et al. 2021
Frequency	Weekly sessions = less than once weekly	Krouwel et al. 2021
Type of hypnosis	modern hypnosis > mixed form > classical hypnosis	Flammer & Alladin 2007
Self-hypnosis	Therapist delivered + self-hypnosis > self-hypnosis only	Chen et al. 2017
	Therapist delivered + self-hypnosis > therapist delivered only	Milling et al. 2018
Direct suggestions	direct suggestions used = no direct suggestions used	Milling et al. 2021
Standardization	Standardized = not standardized	Holler et al. 2021
Hypnotizability	Higher hypnotizability → better outcome	Flammer & Bongartz 2003; Flammer & Alladin 2007 Milling et al. 2021
**Characteristics of the control group**		
Type of control group	TAU = attention control	Tefikow et al. 2013; Holler et al. 2021; Schnur et al. 2008

Characteristics of the primary studies		
Sample size	Smaller sample size → better outcome	Schnur et al. 2008
Country	Studies conducted in Europe > America	Chen et al. 2017

TAU, treatment as usual/standard care; > significant subgroup effects reported; = no significant differences between subgroups; → significant correlation.

Supplementary Figure 1. Sankey diagram of primary study overlap

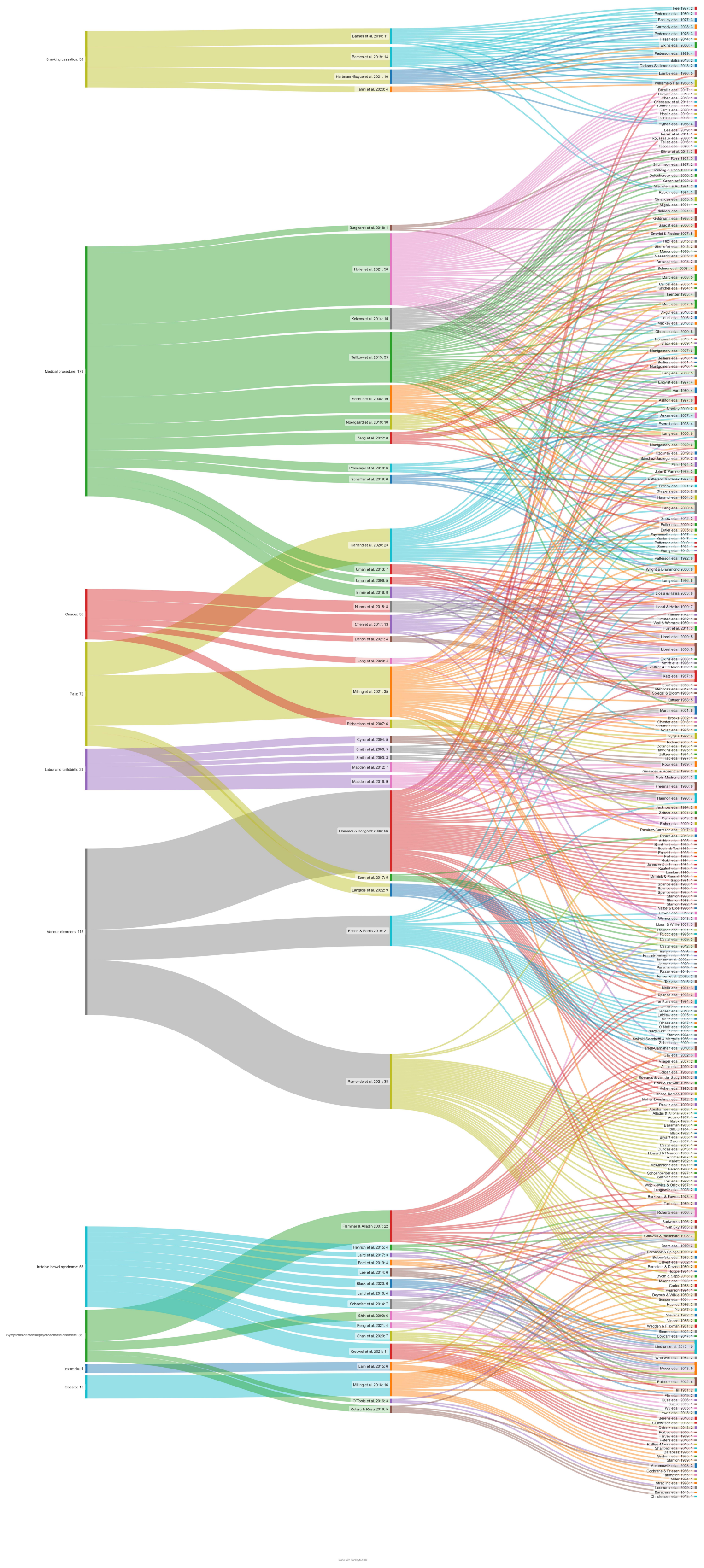

Supplementary Figure 2. Summary of AMSTAR 2 quality assessment of the included reviews

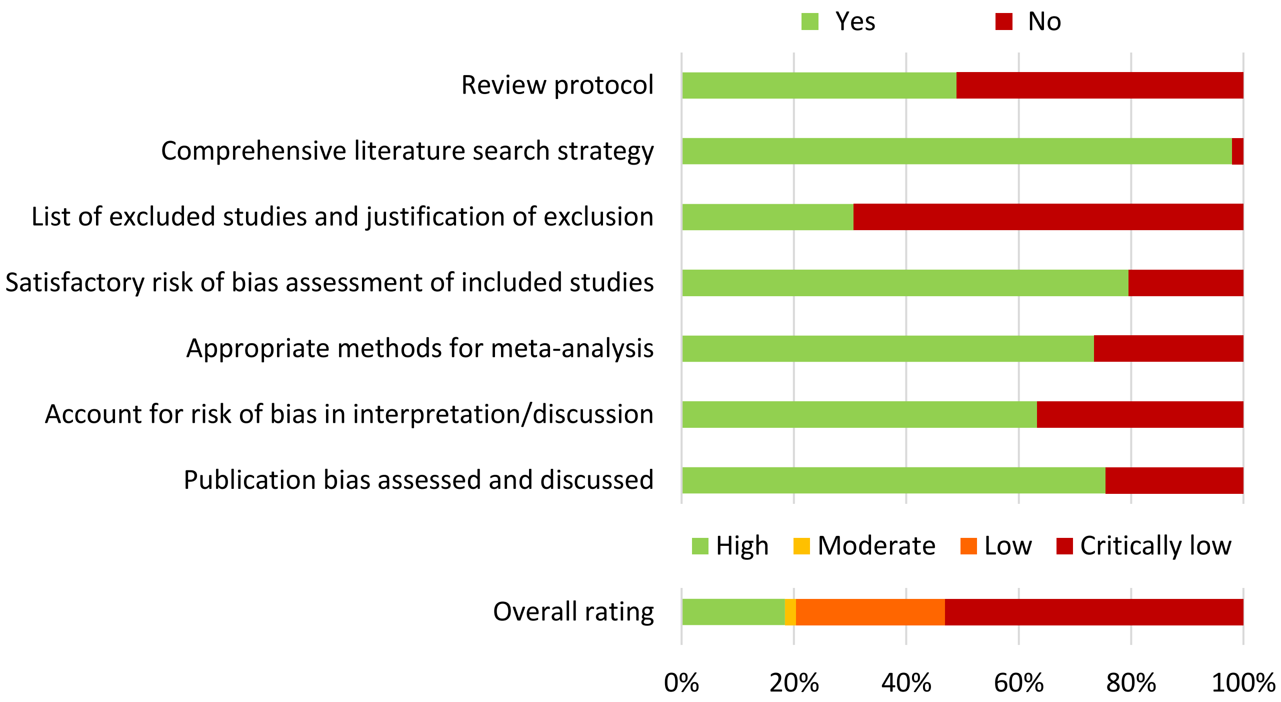

Supplement: Supplementary file 4 [file Data_Sheet_2.PDF]
